# Accurate Protein Structure Annotation through Competitive Diffusion of Enzymatic Functions over a Network of Local Evolutionary Similarities

**DOI:** 10.1371/journal.pone.0014286

**Published:** 2010-12-13

**Authors:** Eric Venner, Andreas Martin Lisewski, Serkan Erdin, R. Matthew Ward, Shivas R. Amin, Olivier Lichtarge

**Affiliations:** 1 Department of Molecular and Human Genetics, Baylor College of Medicine, Houston, Texas, United States of America; 2 Graduate Program in Structural and Computational Biology and Molecular Biophysics, Baylor College of Medicine, Houston, Texas, United States of America; 3 W. M. Keck Center for Interdisciplinary Bioscience Training, Houston, Texas, United States of America; King's College London, United Kingdom

## Abstract

High-throughput Structural Genomics yields many new protein structures without known molecular function. This study aims to uncover these missing annotations by globally comparing select functional residues across the structural proteome. First, Evolutionary Trace Annotation, or ETA, identifies which proteins have local evolutionary and structural features in common; next, these proteins are linked together into a proteomic network of ETA similarities; then, starting from proteins with known functions, competing functional labels diffuse link-by-link over the entire network. Every node is thus assigned a likelihood *z-*score for every function, and the most significant one at each node wins and defines its annotation. In high-throughput controls, this competitive diffusion process recovered enzyme activity annotations with 99% and 97% accuracy at half-coverage for the third and fourth Enzyme Commission (EC) levels, respectively. This corresponds to false positive rates 4-fold lower than nearest-neighbor and 5-fold lower than sequence-based annotations. In practice, experimental validation of the predicted carboxylesterase activity in a protein from S*taphylococcus aureus* illustrated the effectiveness of this approach in the context of an increasingly drug-resistant microbe. This study further links molecular function to a small number of evolutionarily important residues recognizable by Evolutionary Tracing and it points to the specificity and sensitivity of functional annotation by competitive global network diffusion. A web server is at http://mammoth.bcm.tmc.edu/networks.

## Introduction

Similar proteins typically perform similar functions. Nevertheless, defining the nature, extent and mix of which similarities bear most directly on function remains a challenge [1]. This problem is acute even for proteins with solved structures such as those targeted by the Structural Genomics project [2], [3]. Many of these were specifically chosen to have little or no homology with proteins that were already characterized functionally and about one third (or 3002 out of 9122) still lack known function. More sensitive methods to recognize functionally relevant similarities are therefore needed that also take care not to increase false annotations that may arise whenever protein homologs diverged in function [4], and which may then propagate through further rounds of computational annotations [5], [6], [7]. Therefore it remains pivotal to define measures of protein similarity that are highly functionally relevant, and then to devise analysis techniques that draw correct functional inferences from them.

Currently, a great diversity of protein similarity measures are used to infer functions. They include sequence homology [8], [9], [10], phylogenetic ancestry [11], [12] substrate similarity [13], co-expression [14], [15], physical interaction [15], [16], [17], genetic interaction [18], [19] or analogies of sequence [20], [21] or structure motifs [22]–[27]. Some methods compare divergent, aligned proteins to spot discriminating residues that suggest functional signatures in sequences (EFICAz2) [20] or in structures (FLORA) [23]. Since relatively few of all sequence or structure variations are necessarily functionally relevant [28], [29], other methods focus on just a few but presumably key residues. For example, residues could be taken from concave protein regions since these are more likely to be functionally important (pevoSoar) [22]. More generally, residues can be taken from putative functional sites in their relative three-dimensional (3D) configuration to create 3D templates: composite structural motifs of a few amino acids that directly mediate function. Experimentally validated 3D templates are available but they do not cover all of functional space [30], however, and their simple geometric matches can be spurious [31], [32]. Profunc [33] mitigates these problems by using multiple template sources, including enzyme active sites gathered from the Catalytic Site Atlas [30], ligand and DNA binding sites [34], and automatically generated triads of amino acids called ‘reverse templates’ [33]. None of these methods begin with a dedicated approach to identify protein functional sites and their key determinants.

Here, we focus on 3D templates that embody evolutionary information. Our rationale is that functionally important residues may often be distinguished by sequence variations that correlate tightly with evolutionary divergence [35], [36] and form spatial clusters in structures [37], [38], [39]. Such clusters then suggest binding sites or catalytic sites on the surface, and allosteric pathways internally [35], [37], [40], [41], [42], [43] which, in turn, efficiently guide experiments to block, separate, rewire, or mimic function [44]–[54]. Building on these computational and experimental studies that demonstrate evolutionary identification of functional determinants, our approach ranks the relative evolutionary importance of every residue in a protein sequence with the Evolutionary Trace [35], [55] (ET), and then selects the six most important and clustered surface residues to define a 3D template. The geometric matches of these evolutionary templates in other protein structures at sites that are themselves evolutionarily important then define Evolutionary Trace Annotation (ETA) annotations [31], [56]. So far, ETA annotations have been shown to be functionally specific (positive predictive values above 90%) in enzymes and non-enzymes alike [57], but their functional resolution and coverage are limited. For example, enzyme predictions, are at the third rather than the fourth EC level and coverages range from 40 to 70 percent.

We hypothesize that we can improve performance by basing annotations not only on direct matches to a protein of interest, but on all ETA matches detected across the proteome. This follows studies that systematically pool together multiple functionally-relevant matches between proteins [58]. One such approach examines a protein-protein interaction neighborhood to choose predictions that maximize the observed versus expected frequency of a function in a local subnetwork [59]. An extension further factors the network's topological weights into the prediction [17]. Another approach employs a probabilistic analysis of the functional neighborhood defined by sequence alignment bit-scores [60]. More generally, a network in which each node is a protein and each edge is a pairwise match can be constructed and then analyzed with clique or module detection algorithms to increase the amount of information involved in a prediction. For example, Mcode uses a greedy algorithm to grow clusters from a seed node [61]. SpectralMod iteratively cuts edges until only dense clusters remain [62]. CFinder groups together tightly connected cliques into clusters [63]. Once a cluster is identified, enriched functions in that cluster are propagated to unannotated members of the cluster [64]. There is, however, no single best clustering method for all topologies, and clustering approaches can be less accurate than local methods [65].

Global graph theoretic tools may further improve performance by including the entire network topology into annotation transfer [64]. One such approach optimizes the labeling of proteins with unknown function in order to maximize connections between proteins with the same function [16]. Other methods use Markov random fields and assume that the function of every protein in the network is dependant, in probabilistic terms, only on its direct neighbors [66]. Lastly, flow or diffusion-based methods have been proposed that are able to take advantage of both global structure and local similarity. FunctionalFlow is an iterative algorithm that simulates the flow of liquid through a network for the purposes of functional annotation [67]. These methods can also be directly optimized and have been applied to prediction of protein-protein interactions [68] as well as prediction of Gene Ontology (GO) terms [69], [70].

Therefore, we applied a global network diffusion method to integrate all ETA matches into the functional inference. Since most functions permeated and reached most of the protein nodes, we further devised a *z*-score statistic of confidence for every protein-function pairing. The function with the greatest confidence was then chosen as the most likely annotation for that protein. In practice, the correlation between confidence and accuracy is strong. As a result, the ETA network diffusion method yields accurate predictions at the fourth, and highest resolution, level of the Enzyme Classification and also substantially reduces false positives compared to other annotation methods.

## Results

**Figure 1:**
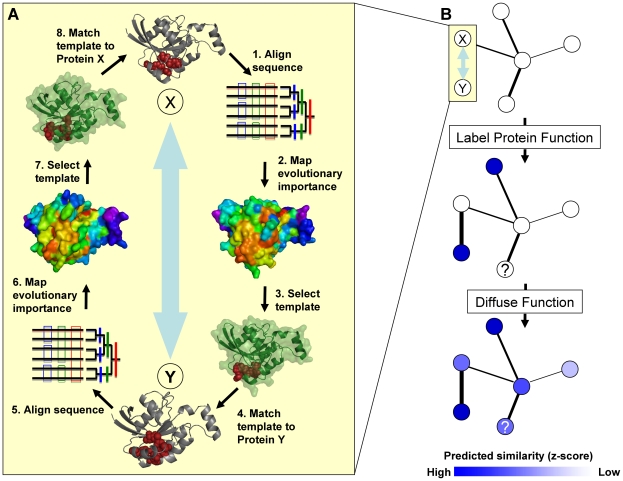
Overview of ETA Network Diffusion. 1A. We detect similarities between proteins using Evolutionary Trace Annotation (ETA), which consists of three steps. First, the Evolutionary Trace (ET) algorithm ranks positions in aligned sequences by the correlation of their variations with evolutionary divergence. These ranks of evolutionary importance are mapped onto the protein structure. Second, six amino acids are selected heuristically based on their evolutionary importance, proximity and surface exposure, forming a structural template (red spheres). Third, the template is matched against proteins with known function. These steps are repeated for the matched proteins in order to verify that the match is reciprocal. Significant matches are selected by an SVM (not depicted). 1B. We construct a graph using ETA matches so that nodes represent protein chains and edges represent evolutionary and structural similarity. We select an enzymatic function and apply one of three labels to every node in the network: blue if the node is known to have that function, white if it is known to not have that function, or “?” if it is unknown whether or not the node has that function. We then allow these labels to “diffuse” to all other nodes in the network based on the strength and number of connections. This results in a weight assigned to every node for all enzymatic functions present in our network. In a final step (not depicted) we normalize the weights assigned to a particular node with respect to all other un-annotated nodes in the network. The normalized weights (called z-scores) are compared. The functional label with the highest z-score is taken as the prediction, and the magnitude of the z-score is used as a measure of confidence.

Annotation by ETA network diffusion proceeds in two main steps: the construction of a network, described here and in Figure 1A, followed by the diffusion of functional labels, described next and in Figure 1B. First, the Evolutionary Trace (ET) algorithm [35], [55] ranks the evolutionary importance of every residue in a protein sequence by correlating their variations with phylogenetic divergences. Top-ranked residues (usually defined as those in the top 30^th^ percentile) are then mapped onto the structures, where typically they cluster spatially at the locations of functional sites. Second, ETA heuristically selects six top-ranked, proximate, surface amino acids from each protein to define a 3D template. Third, it searches all other structures in the dataset for geometric matches to this template. Since often geometry is insufficient to generate specific matches, ETA also specifies, with an SVM, that the matched sites should themselves be composed of functionally important (top-ranked) residues. Fourth, and last, it narrows the list to reciprocal matches – i.e. those cases in which protein A has a significant match to protein B and protein B has a significant match to protein A. In our ETA networks, it is these reciprocal matches that are turned into weighted edges by averaging the ET score (which indicates evolutionary similarity of the match) and the RMSD (which indicates structural similarity of the match, see Methods). Thus, the network nodes represent individual protein structures, and the network edges represent molecular and evolutionary similarities identified by ETA [57] that, by our hypothesis, should bear directly on the potential for proteins to have identical biochemical roles.

Next, once the network has been constructed, a diffusion mechanism lets annotation data flow from node to node so that the poorly annotated regions draw information from the richly annotated ones.

Formally, let a graph have *n* nodes, each depicting a protein, such that a number *p≤n* carry labels *y*_i_ that represent specific functions, where the index I ranges from 1 to n. ETA matches provide all-against-all linkage data on by averaging the ET score and RMSD of the template match to define edges in a symmetric adjacency matrix, *w*. If nodes *I* and *j* have a mutual ETA template match (Figure 1A), then they are linked by edges so that the adjacency matrix entry is set to *w*_i*j*_ *>*0, otherwise *w*_i*j*_ *= *0 if there is no known or significant similarity. The problem is then to infer function for the remaining *q* = *n - p* nodes from the nodes with known labels *y* and the network's connectivity. This frames an optimization problem between two conditions: (i) that the function of neighboring nodes be similar (smoothness condition), and (ii) that the final label of a node be consistent with its initial label if its function was known (initial condition). Both conditions can be most simply represented by a quadratic “cost” function [71]:
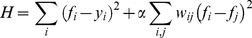
(1)where the functional label *y_i_* is set to either 1 if node *I* has a function y, to −1 if it does not have that function, and to 0 if there is no evidence either way (unlabeled node); and where −1≤*f_i_*≤1 are the predicted functional labels of the output prediction vector. Finally, the coupling parameter α is analogous to a diffusion coefficient that balances smoothing with loss of the initial labels. This formulation is empirical, but it is convenient because solving *f = *{*f_1_,*…,*f_n_*} is equivalent to solving a sparse linear system with the graph diffusion kernel, *viz.* 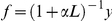, where 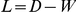
is the Laplacian matrix, with the link weight matrix 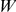 and 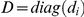, 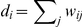. In the more limited context of binary protein function classification the computational efficiency of this approach surpassed semi-definite programming (SDP) and support vector machines (SVM) [72].

Here, in order to extend this method to multi-label classification we need to account for the bias introduced by different label frequencies. To do this, we introduce a prediction *z*-score, defined by the solution 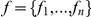 as 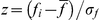, where 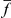 denotes the average and 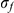 the standard deviation evaluated on all unlabeled nodes; *z* measures the positive deviation from the expected random mean in standard deviation units and thus eliminates any absolute bias due to initial conditions in *y*, which then allows a meaningful comparison between multiple functional classes of variable size within the network. After diffusing every function to every node, we use the function with the highest z-score as our prediction. In this way, we add a new step to the ETA process, coupling relevant and non-trivial edge detection with global function propagation.

We have benchmarked this method on two distinct test sets. First, we perform a leave-one-out test on the FLORA [23] test set in which we attempt to predict the function of a given protein using matches to the remaining proteins in the test set. Second, we have assembled a test set of Protein Structure Initiative [3] (PSI) proteins, and attempt to predict their function by matching them against a representative subset of the Protein Data Bank [73] (PDB). Finally, we examine the effect of lowering availability sequence similarity, an important consideration because the PSI often contains structures with few homologs [74].

### Comparative study against the FLORA method

**Figure 2:**
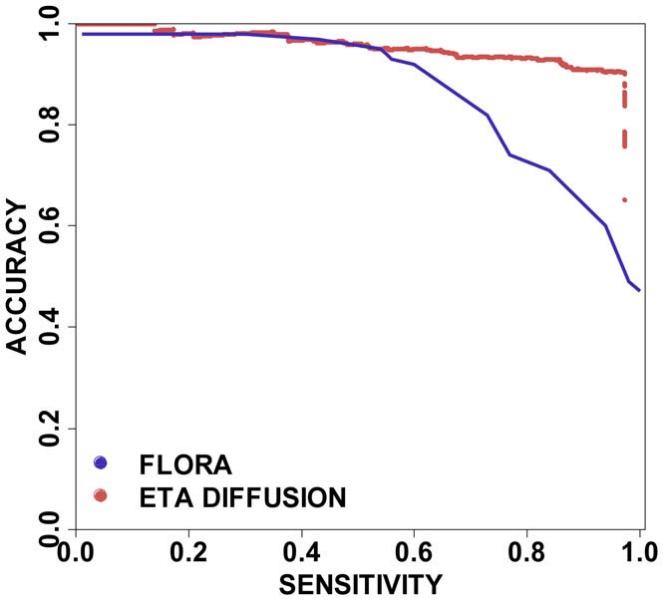
Performance on the FLORA test set. The diffusion method shows a clear improvement at higher sensitivities.

Like ETA network diffusion, FLORA [23] is a structure-based annotation method; it is recent, and thus provides a state-of-the-art baseline control. We compared both methods in a leave-one-out experiment against FLORA's published dataset and 3 EC digit prediction results (Figure 2). Those predictions were stratified by stepping down through each z-score and, at each threshold, calculating the cumulative accuracy, coverage and sensitivity. Any true prediction with a z-score that fell below the threshold was deemed a false negative (fn) while those that fell above the threshold were counted as either true (tp) or false positives (fp) depending on whether they matched the known function. Sensitivity (calculated as (tp/(tp + fn)) rose, and prediction confidence fell, as the *z*-score threshold was lowered.

Overall, the ETA network diffusion markedly reduces false positives. Initially, like FLORA it maintains perfect accuracy up to 14% sensitivity, as shown in Figure 2, and its accuracy decreases to a similar extent until 54% sensitivity. Thereafter, however, the two methods diverge: accuracy drops steadily for FLORA but not for ETA network diffusion. The difference is largest near 97% sensitivity where ETA network diffusion has 4-fold fewer false positives (90% *vs* 60% accuracy). Finally, past 97% sensitivity, incorrect predictions for ETA Network diffusion soar, but this is expected from the extremely low confidence of the predictions past that point (the *z*-score is below −0.05). Since FLORA [23] was itself superior to other template methods, such as CATHEDRAL [75] and Reverse Templates [76], these results suggest that ETA network diffusion also outperforms these approaches.

### Structural Genomics

Next, we sought to test ETA network diffusion on a larger and more realistic test set of annotated structural genomics proteins (Structural Genomics test set). These proteins were added to an ETA network of a representative subset of the PDB (PDB 90, non-redundant at the 90% sequence identity level) by identifying ETA matches between proteins in the test set and proteins in the PDB 90.

**Figure 3:**
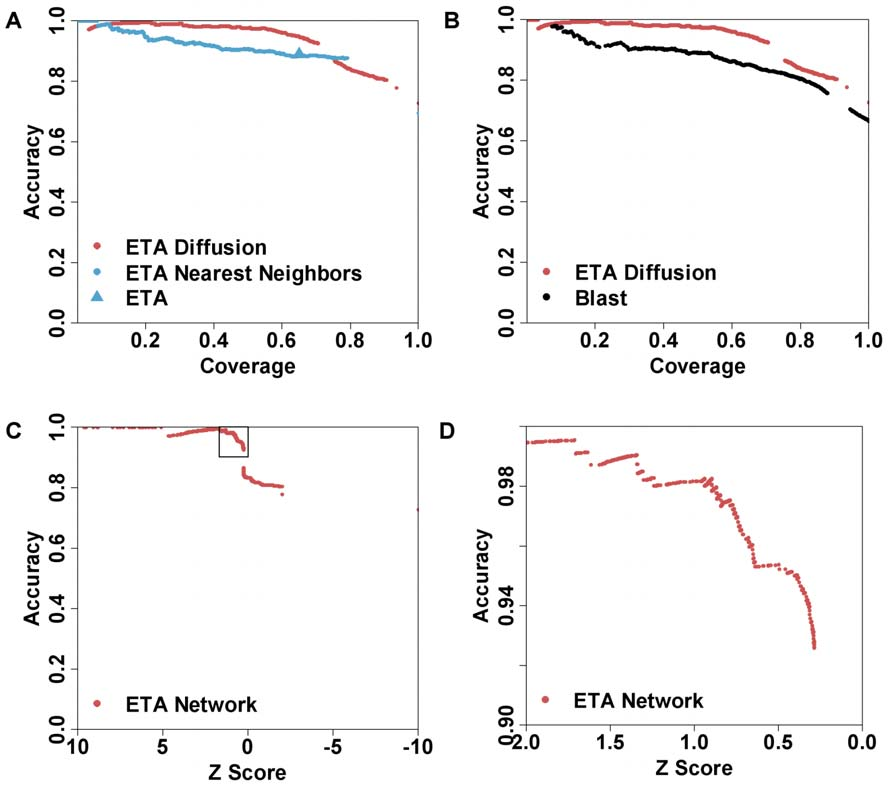
4 EC Performance on Structural Genomics test set. 3A. Accuracy/coverage tradeoffs of ETA network diffusion and nearest neighbors are shown in red and blue circles, respectively. Coverage (percentage of entire test set) increases as confidence decreases, so at 10% coverage we show the accuracy (# of true predictions/# of predictions made) of our 10% most confident predictions. Blue triangle shows performance of ETA. Diffusion gives clear accuracy advantages at most coverage cutoffs. 3B. Performance compared to the top match from a BLAST search of Swiss-prot. Diffusion on an ETA network clearly outperforms BLAST (black circles) at most coverages on this dataset, demonstrating the need for complementary structural based methods. 3C: Accuracies when the z score cutoff is varied. For each z score, we plot the accuracy of all predictions with that score or higher. Accuracy and z score show a positive correlation. Accuracy shows a steep decline after z = 0.4. 3D shows a magnified view of the beginning of the steep decline.

The ETA network diffusion proved equally accurate on this test set as it had before on the FLORA test set and the confidence *z*-score continued to separate the reliable predictions from those that were not. Thus at the highest resolution EC digit, the fourth level that typically indicates the substrate of an enzyme, the accuracy was better than 98% up until 45% coverage corresponding to a *z*-score of 0.89 (Figure 3A). It then decreased slowly to reach 96% at 60% coverage, corresponding to a *z*-score of 0.68, and then it dropped slightly more rapidly thereafter. Compared to the single point for ETA, at 65% coverage, the network achieved 6% better accuracy and halved the false positives (36 *vs* 74).

The specificity may be increased further at the expense of the functional resolution: at the third EC digit (Figure S1A) the accuracy remains perfect (100%) up to 29% coverage, which corresponds to a z-score of 1.7 or above, and it remains over 98% until 72.7% coverage and a z-score of 0.67 or above. The accuracy gain over ETA is nearly 4%, at 73% coverage, which translates to 2.5-fold fewer false positives (19 *vs* 53).

### Impact of network diffusion

In order to distinguish the contribution of global network diffusion from the contribution of purely local matches, we compared these results to a nearest neighbor algorithm on the same underlying network (Figure 3A). Diffusion proved more accurate until the low z-score of 0.3, which corresponds to 70% coverage. These gains can be striking. For example, in the region near 50% coverage, diffusion is 7.7% more accurate, representing 4-fold fewer false positives (12 vs 48). Likewise, this pattern is repeated for 3 EC digit annotations (Figure S1A). At 50% coverage, diffusion has 2.5-fold fewer false positives (7 *vs* 19) and it is interesting to note that the crossover point is identical, with z-scores both near 0.3, which in this case is 80% coverage. Thus, nearest neighbors is less accurate than global network diffusion at all but a small coverage range, which is properly identified by low z-scores. Since the only additional information available to the diffusion method is global network information, i.e. information not available in matches in the local neighborhood, we conclude that the global topology of the network provides important annotation information and this information is tapped into by the diffusion mechanism embodied in the cost function (eq. 1). In that regard, Figure S4C shows that, in cases where a path exists between proteins, a significant number of proteins with identical functions are removed from each other by more than 1 link (42.9%), 2 links (32.2%) and even 3 or more links (26.5%).

### Structural Genomics annotations *vs* BLAST and PSI-BLAST

Likewise, it is also important to examine whether structural genomics data, combined with ET network analysis, provide extra information over purely sequence-based methods. In order to compare the accuracy/coverage with BLAST [9], the most widely used tool for functional annotation [77], a BLAST search for each protein in the structural genomics test set against Swiss-Prot [78] was followed by comparing the accuracy to ETA network diffusion. We then repeated this process using PSI-BLAST. BLAST proved to be more accurate on this testset than PSI-BLAST, likely due to the availability of homologs in Swiss-Prot. Swiss-Prot contains vastly more potential matches than the PDB90, likely biasing this comparison in favor of BLAST. Despite this, ETA network diffusion had a consistent 4% accuracy advantage for 3 EC digit annotations over BLAST down to a z-score of 0.3, or 81% coverage (Figure S1B). This corresponds to a 4-fold reduction in false positives (7 vs 28).

Importantly, at a higher functional resolution (4 EC level annotation), the gains are even more impressive (Figure 3B). The accuracy is 9% better at 50% coverage and there is no crossover point: the accuracy of ETA network diffusion is never exceeded by the accuracy of BLAST. This corresponds to a nearly 80% reduction in false positives (12 vs 57). Thus, the more precise the annotation, the better ETA structural genomics networks perform relative to sequence comparisons.

### Accuracy and confidence scores

To assess whether the confidence *z*-score can reliably identify false predictions, we examined the correlation between accuracy and the z-score. The trends are similar for both 3 (Figure S2 C&D) and 4 EC digit (Figure 3 C&D) predictions: the accuracy is nearly perfect for *z*-scores above 2, it drops slightly between 2 and 0.4, then it steeply declines thereafter to level out towards 87% and 81% accuracy for 3 and 4 EC digit predictions, respectively.

In practice, of over 649 4 EC level predictions that fell above a *z*-score threshold of 0.5 in the structural genomics test set, only 30 were predicted to have a function different from the one listed in the PDB. Assuming the latter are correct, this yields a rate of accuracy of 95.4%. Likewise, 382 predictions fell over a z-score of 1, and of these 375 matched existing reference annotations, yielding a 98.2% accuracy.

### *Staphylococcus aureus* case study

The ETA network diffusion method was then applied to a set of 2767 structural genomics proteins categorized in the PDB with “unknown function”. Matching to the PDB90 at a *z*-score cutoff of 0.5 led to 257 predictions; however, not all of these predictions were completely novel since some of the PDB profiles did contain some functional inferences.

**Figure 4:**
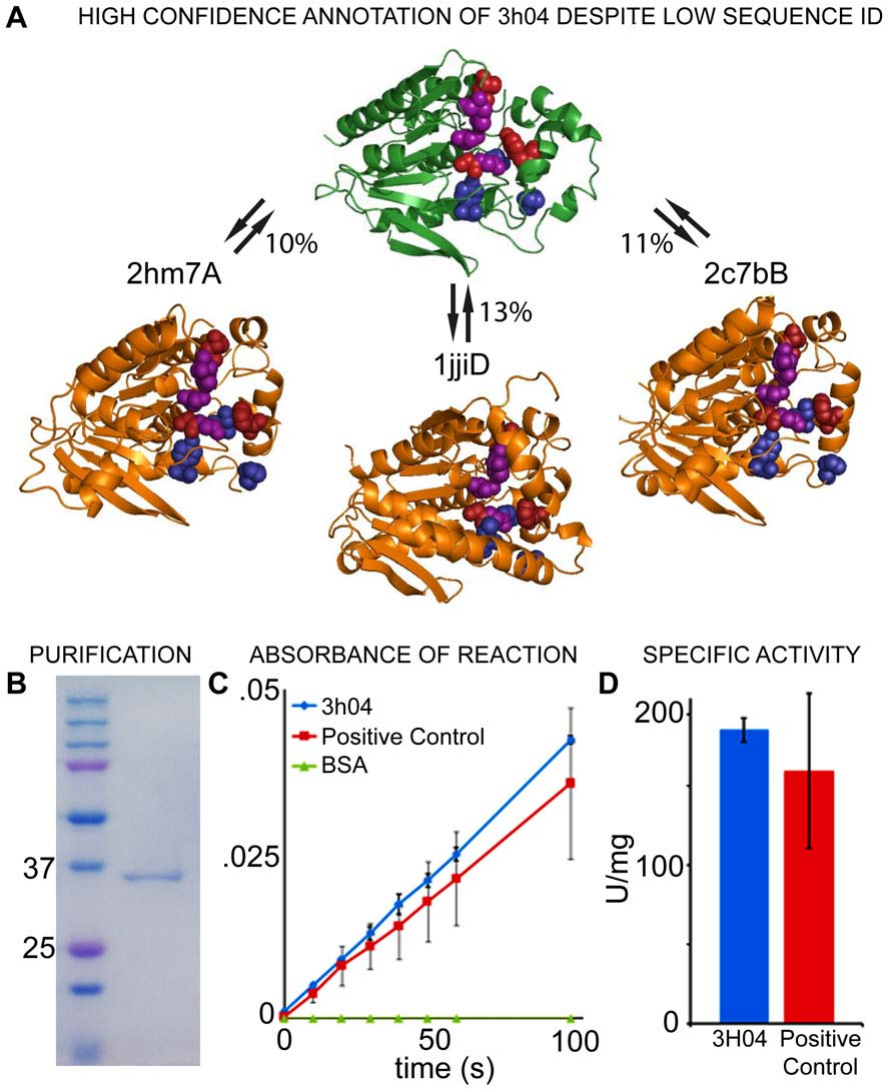
*in vitro* biochemical assay confirms the ETA network diffusion prediction of 3h04 as a carboxylesterase. A) The prediction of carboxylesterase function for this unknown protein is based on ETA template matches to three chains, all of which have identical function and fold, and low sequence identity with the query protein. B) 10 µg of purfied 3h04 was run on a SDS-12% polyacrylamide gel and stained with Coomassie brilliant blue. The single band shown at 35 kDa corresponds to his-tagged 3h04. C) Plot of absorbance at 405 nm vs time for 3h04 (blue), esterase from porcine liver (Sigma, red), and BSA (Sigma, green). D) The specific activity of 3h04, 193±8 (blue), is similar to that of the esterase from porcine liver, 166±51 (Sigma,red). Specific activity is represented in Units (U) per mg of protein. All error bars depict standard deviation.

As an illustration, our method confidently (z score over 2.9) predicts carboxylesterase activity (EC3.1.1.1) for a bacterial protein with unknown function (PDB 3h04 chain A). The protein (Uniprot accession Q99WQ5, gene name SAV0321) originates from a vancomycin drug resistant strain of the bacteria *Staphylococcus aureus*, an organism that can cause life-threatening infections [79]. This annotation, shown in Figure 4A, is based on template matches to three chains which share carboxylesterase activity and range between 10 and 13% sequence identity with the query chain. The three matches are chains 2hm7A, 1jjiD and 2c7bB, which all belong to a Rossman fold (CATH [80] fold description 3.40.50.1820), although the query protein lacks fold annotation. A PSI-BLAST [10] search finds many unannotated homologs in other strains of S. aureus as well as several other bacteria, such as *Enterococcus faecalis* and *Lactobacillus buchneri*. The first-EC-level classification hydrolase (EC 3) and second-level classification esterase (EC 3.1) can begin to be gleaned from matches above BLAST e-values of 9e-10 and 6e-06 respectively. Annotations that disagree with ours, such as arylesterase (EC 3.1.1.2) are first matched at BLAST e-value of 0.003. Homology with a carboxylesterase is first found in a match with BLAST e-value of 0.004. The sequence motif-based EFICAz2 [20] method makes no prediction, thereby confirming that this protein is difficult to annotate from sequence information alone.

The DALI [81] algorithm, which performs whole domain three-dimensional structural alignments, reveals similarities to the same carboxylesterases that ETA matches. The catalytic triad of chain 2c7b is known to be Ser154, Asp251, and His281 [82], and these residues are aligned with a corresponding serine, aspartic acid, and histidine in chain 3h04A, suggesting functional importance for these residues. All three residues of this triad were included in the reciprocal ETA template.

In order to definitely determine if SAV0321 possesses carboxylesterase activity, *in vitro* biochemical techniques were performed next. A his-tagged version of SAV0321 was expressed in E. coli and purified by affinity chromatography (Figure 4B) and its ability to hydrolyze the carboxylesterase substrate 4-nitrophenyl acetate to form 4-nitrophenol and acetic acid was measured. The production of 4-nitrophenol is detectable by UV spectrometry at a wavelength of 405 nm (Figure 4C) [83], [84] From the absorbance values we extrapolated the specific activity (Figure 4D), and we showed that it is similar to the positive control, a carboxylesterase from porcine heart (Sigma). Moreover, this specific activity was also consistent with specific activity values of other known carboxylesterases for the same substrate [85], [86], [87]. BSA, the negative control, has no such activity. Therefore, we can conclude that the ETA network prediction of carboxylesterase activity for SAV0321 is correct.

### Sequence similarity

**Figure 5:**
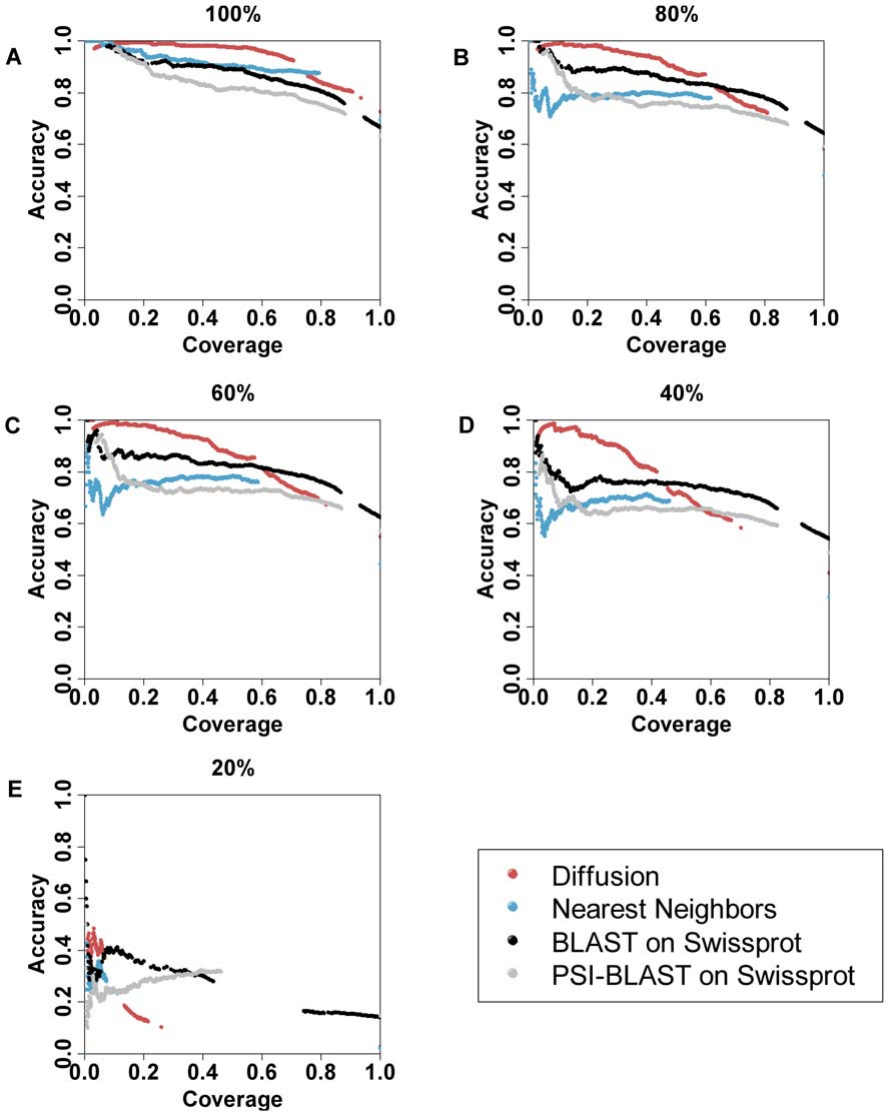
Performance penalty as edges are removed from a graph according to the sequence similarity of the nodes they connect for 4 EC predictions. Accuracy/coverage tradeoffs of ETA network diffusion, nearest neighbor, and the top match from a BLAST search against Swiss-prot are shown in red, blue and black circles respectively. Coverage increases as confidence decreases, meaning at 10% coverage we show the accuracy of our 10% most confident predictions. Maximum allowed sequence identity is 80% in 3A, 60% in 3B, 40% in 3C and 20% is 3D. Accuracies decline with each removal, but ETA network diffusion maintains higher accuracy at high confidences/low coverage.

In order to test the impact of homology on these predictions, edges in the structural genomics test network were removed, in increments of 20% sequence identity, to eliminate links between any two proteins with more than 80% sequence identity, then with more than 60%, and so forth. This creates new networks with ever less information due to homology (Figure 5). At and above 40% sequence identity, accuracy of ETA network diffusion exceeds BLAST's by between 7% and 8% in the low coverage, high confidence region of the plot (below 50% coverage). Thus, ETA predictions depend less on sequence identity than BLAST. Of note, a BLAST search against the SwissProt database biases the results in favor of BLAST, since it contains more than 27 times [78] the number of sequences than there are structures in our PDB-derived dataset. Despite this, ETA network diffusion gives accuracies higher than BLAST in the high confidence interval, demonstrating the effectiveness of the *z*-score for distinguishing the correct predictions, even when faced with less reliable matches.

In practice, most proteins will either a) exhibit very high sequence identity to a close homolog, or b) will not exhibit homology to any protein, though there are a significant number that lie somewhere in between (Figure S3). Therefore, the cutoffs at 100% and 20% in Figure 5A and 5E represent the most likely scenarios for protein annotation. In both cases, ETA network diffusion outperforms BLAST and nearest neighbors. With the cutoff set at 100% sequence identity, ETA network diffusion has a clear advantage at most coverages, as we have seen. At 20% sequence identity the accuracy of all methods is low (below 40%). However, ETA network diffusion maintains a small accuracy advantage among the very highest confidence predictions (below 7% coverage). Thus, at levels of sequence identity likely to be found in practical test cases, ETA network diffusion maintains an accuracy advantage.

## Discussion

This work shows that the diffusion of protein functions over a network of local structural and evolutionary similarities yields accurate functional predictions. The key distinguishing features of the diffusion process are (1) that it is guided by functionally-relevant links. These links are defined by reciprocal ETA matches, which establish that two proteins share some key functional determinants that are in identical structural configurations. Importantly, they can be generated from ET analysis without any prior knowledge of a protein's likely function or mechanism. However, these links do reflect both evolutionary and structural information about the most functionally relevant parts of a protein. (2) Network diffusion also puts every link and every known prior annotation on par across the entire network, so that all annotations compete without bias, and the best one at each node is objectively assessed with a statistical *z*-score.

Compared to other state-of-the-art approaches, confidence values proved better at sorting unreliable predictions, and in turn this improved annotation accuracy at the third and at the fourth, and most specific, EC levels. These results are general since they apply across all types of enzymes, and they are accurate since false positive rates decrease substantially—between 2- to 5-fold. The many predictions on unannotated proteins demonstrate the benefits of the repeated use of evolutionary information and its integration with structural information over the structural proteome.

In order to identify the various sources of information that improve annotation we compared the impact that negative information has on diffusion on identical PDB 90 networks and Structural Genomics testsets. Accuracy-coverage curves with negative labels (Figure S4A, red) have a substantial accuracy advantage over the same curves without negative labels (purple). Without the negative labels, accuracy falls: at 50% coverage it drops by 16% and 10.7% for 3 and 4 EC digit predictions, respectively. Thus, the -1 entries in the *y* vector, which indicate the knowledge that a protein does not perform a specific enzymatic function, contribute significantly to accuracy. By contrast, many annotation methods, for instance the nearest-neighbor and BLAST approaches we benchmark against, do not make use of this information. Hence, knowledge of which proteins in the network lack a particular function is critical for function prediction with diffusion, and may contribute to accuracy advantages over other methods.

Additionally, in order to assess the contribution of distant positive labels to annotation, we examined the shortest path lengths between proteins with the same and differing functions. Figure S4C shows a stacked histogram comparing the lengths of shortest paths in the network between nodes with a correct prediction and nodes in the dataset, segmented by the confidence z-score. Proteins with the same function (blue) tend to have shorter distances between them than proteins with different function (orange), indicating that functions generally cluster in the network. However the distributions have long tails, especially for predictions with a z score less than 3, so that in a number of instances proteins with the same function can be quite distant. Based on our accuracies, the diffusion process is presumably able to connect these distant proteins. Therefore, both negative labels and positive labels distant by 10 or more nodes are additional information sources that contribute to more accurate predictions in ETA Network Diffusion.

Strikingly, these results rely on the large-scale comparison of just six evolutionarily important template residues, chosen protein by protein. The accuracy of the network shows that these residues effectively capture the determinants of protein function. This in essence, validates on a large scale the notion that ET analysis identifies key functional residues—consistent with the conclusions of many experimental case studies [36]. Notably, as this study draws from many previous ideas, such as 3D templates [57], [76] evolutionary importance [35], functional site analysis [88], molecular determinants of function [23], [20] and network analysis [64], [69], [89], [90] it combines them uniquely by repeatedly relying on evolution at each step of the annotation process.

First, the 3D template residues are selected for their evolutionary importance measured by ET, and for their structural clustering. This local structural motif defined by evolution obviates the need for any prior knowledge, or assumptions, about the nature and determinants of function. This is an advantage since compared to the size of the proteome, there are relatively few proteins with reliable data on the molecular mechanisms underlying their function and specificity, as may be available from the catalytic site atlas [34]. Likewise, these 3D templates also replace searches for structural features, such as clefts, cavities or depressions, which are suggestive but rarely sufficient [88].

Second, the selection of which 3D template matches are functionally relevant also relies on evolution. Out of the profusion of purely geometric matches between a template and protein structures, only those that involve residues with evolutionary importance similar to the template residues are retained. Every accepted match, and therefore every edge in the network, indicates reciprocal similarities of evolutionary constraint and structural context, which raises the likelihood of a true functional similarity.

It follows, third, that diffusion over a network defined by these evolutionary template matches disseminates evolution-guided inferences over the structural proteome. The correlation between the confidence z-score associated with every diffused function and the reliability of annotations confirm that these three hierarchical types of evolutionary inferences—meaning the 3D template, the match, and the diffusion—are all well founded: evolutionary analysis thus dramatically narrows the search for the essential determinants of a protein's function and for their comparison.

**Figure 6:**
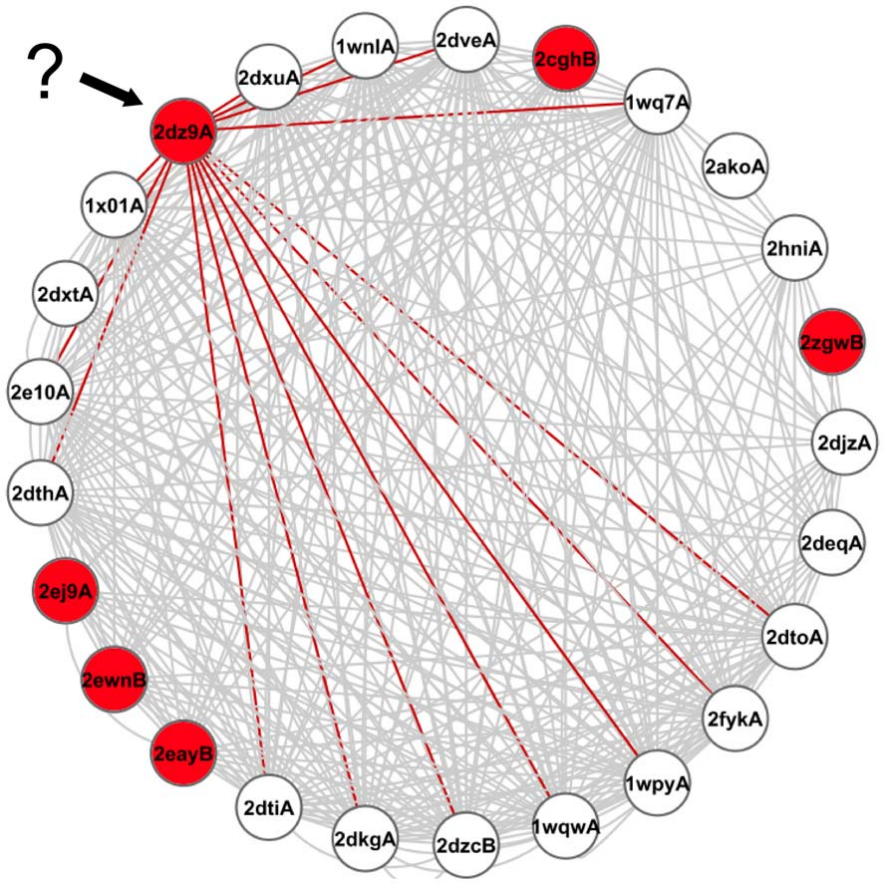
Network neighborhood of PDB structure 2dz9A. Depicts the network neighborhood within 2 steps from structure 2dz9A. Structures in red are annotated as biotin—acetyl-CoA-carboxylase ligases (6.3.4.15). White structures have no function or are part of the test set. The nearest neighbor method leads to no prediction for 2dz9A because all matches are only to proteins without known function, but diffusion leads to a correct prediction because of the proximity to that functional label and high connectivity.

The global network approach also has many intrinsic advantages. It removes the heuristic aspect of the ETA voting approach, [57] it enables global and formal integration of information over the entire structural proteome, and, as a future direction, it prepares the integration of ETA information with many other types of functionally relevant protein similarity, since the latter usually come in the form of pairwise relationships are therefore well suited for network representation [64], [69]. Specifically, the diffusion process is non-local and draws information from all of the functional labels in the network, not just those from direct matches. As a result, it extends prediction coverage compared to strictly local techniques. This is illustrated, for example, by the gene PHO147 in Pyrococcus horikoshii (PDB 2dz9 chain A), as shown in Figure 6. This protein matches solely unnannotated proteins in a well-connected cluster, so both ETA and nearest neighbors can make no prediction. Network diffusion, however, enables more distant annotations to inform the annotation of this node, leading to a correct fourth EC digit prediction.

The computation of a confidence z-score that correlates with prediction accuracy is another contribution of this work. Together with the global diffusion process, it enables unbiased consideration of all possible functions, establishes an objective criterion for selecting the best candidate, and attaches a confidence value to it. As a result, predictions can be stratified by the z-score, yielding the accuracy versus coverage receiver-operator curves, shown in Figures 3C and 3D, that remain close to 100% accuracy over a large coverage. Predictions with a z-score above 2 are over 99% accurate and over a z-score of 1 they are 98% accurate. Conversely, on the FLORA set, the vast majority of false positives also had the lowest confidences (z<−0.05) (Figure 2). The z-score is therefore an adequate marker of confidence with which to recognize unreliable predictions that otherwise would become false positives. Overall, we see from 2 to 5 fold reductions in false positives when compared to ETA, FLORA, nearest neighbors or BLAST.

Collectively these improvements yield confident predictions at the 4^th^ EC level, which identifies precise substrates in many cases. For example, the predicted EC annotation for gene PHO147 in *Pyrococcus horikoshii* (PDB structure 2dz9A) is biotin—acetyl-CoA-carboxylase ligase (EC 6.3.4.15). This function indicates the substrates ATP, biotin and acetyl-CoA-carboxylase, which would not be obtainable from a 3 digit EC annotation (EC 6.3.4, Carbon—Nitrogen Ligases), which usually describes the chemical reaction.

In the future, a number of network diffusion limitations remain to be addressed. Here only enzymatic functions were considered, although ETA itself makes both enzymatic and non-enzymatic predictions using Gene Ontology (GO) terms [57]. The reason was that the network diffusion of labels taken from a GO directed acyclic graph (DAG) is more complex than from the simple EC hierarchy. Another concern is to further extend the coverage of yet unannotated proteins. As seen in Figure 5, ETA network diffusion performs better than a BLAST search when there are fewer homologs at high confidence z-scores. However, many non-homologous proteins share molecular function as a result of convergent evolution, [91] and variations can produce enzymes with similar function but differing sequence motifs [92]. Moreover, enzymatic function can be flexible and depend on context and expression level [7] such that enzymes are promiscuous and may perform several functions [93]. Presumably, to achieve even greater coverage, these problems will need to be addressed by raising the function detection sensitivity of the network. Further improvements in template construction [39], [94] or data integration [69] are possible directions towards these goals.

In practice, the competitive diffusion of Evolutionary Trace Annotations via a global network of local evolutionary and structural similarities provides a highly specific and reliable method to predict the function of novel protein structures. With the goal of minimizing false positives, we showed that the confidence z-score can reliably select correct annotations and identify those that are likely to be false. The improvement over sequence comparison and nearest neighbor methods is most striking for 4 EC level predictions. This leads to 257 high-confidence functional predictions of Structural Genomics proteins (Table S1). For one of these, the prediction of carboxylesterase activity in *Staphylococcus aureus* protein SAV0321 (PDB ID 3h04), we have demonstrated the accuracy of our method through an *in vitro* assay.

## Methods

ETA matches were generated as previously described. [57] Edge weights were built from an ETA match using:
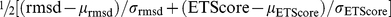
(2)where rmsd and ETScore are output by ETA and describe the template match, µ_rmsd_ is the average rmsd, σ_rmsd_ is the standard deviation of the rmsds, µ_ETScore_ is the average ETScore and σ_ETScore_ is the standard deviation of ETScores. The ETScore describes the difference in evolutionary importance of matched residues and the rmsd describes the difference in structure of the matched template. Network construction and the diffusion algorithm are described in Results.

The nearest neighbor algorithm uses the same underlying network as the diffusion algorithm. It uses all ETA matches to proteins with known function and picks the function with the largest cumulative edge weights. The average edge weight of the winning function is used as a confidence value.

Coverage/accuracy curves were produced by sorting the predictions for the test sets in descending order by z-score, and then plotting a point showing cumulative accuracy (correct predictions/predictions made) and coverage (predictions made/size of test set) for each z-score threshold. The sensitivity/precision plot was produced in a similar manner: test set predictions were sorted in descending order by z-score and cumulative sensitivity (tp/(tp + fn) and precision (tp/(tp + fp)).

Comparison with BLAST was performed by BLASTing each chain in the test set against release 14 of Swiss-Prot, which is contemporary with the PDB data we used. The EC annotation of the resulting homolog with the smallest e value was taken as the predicted function. Comparison with PSI-BLAST was performed in an analogous manner against the same release of Swiss-Prot as the BLAST comparison. PSI-BLAST was allowed to run for 4 iterations.

The Structural Genomics testset was collected from the Protein Data Bank(PDB) by searching for proteins tagged with “structural genomics” and having an EC annotation. There were 1217 proteins with at least a 3-digit EC annotation, and 1036 with a 4 digit EC annotation. The list of candidate proteins for novel predictions were collected by searching the PDB for proteins labeled “structural genomics” and “unknown function.” Structures lacking annotation were counted by searching the protein databank for proteins tagged with “structural genomics” and matching a text search for “unknown function” or “hypothetical.”

The PDB 90 dataset was downloaded from the PDB website. Of approximately 18,600 proteins, 17,924 of them had enough homologs to perform an Evolutionary Trace. Annotations from the PDB were supplemented with annotations retrieved from the GOA database [95].

In the FLORA testset there were 911 domains which represent 821 unique PDB chains. Of those, Evolutionary Trace (ET) was able to find enough homologs for 806, which we used in our leave-one out experiments. The 15 proteins for which ET was not able to produce results are reflected in the lack of perfect sensitivity in Figure 2. ETA predictions of three-digit EC functions were made as described previously.

ClustalW [96] was used to calculate sequence identity between matches found between the testset of 1217 structural genomics proteins and their BLAST matches in SwissProt. Only matches with EC annotation were recorded. 10 sets of predictions were created, one for each {100, 90, 80, 70, 60, 50, 40, 30, 20, 10}% sequence identity cutoff. At each threshold we ignored matches that exceeded the allowed sequence identity. For BLAST predictions, the match with the lowest e-value below the given sequence identity threshold was used. Self matches were excluded.

For the *S. aureus* case study, the EFICAz^2^ search was done through the PSiFR [97] tool provided at http://psifr.cssb.biology.gatech.edu/. The PSI-BLAST search was done through the web interface at http://www.ebi.ac.uk/Tools/psiblast/.

The structural comparisons were done with the DALI web interface [81]: http://ekhidna.biocenter.helsinki.fi/dali_server/.

Network distance calculations were performed via the networkX python library.

### Cloning and Expression

3h04 was amplified by PCR using the following primers: 5′- CTCCGTCGACAAGTGACTGAAATTAAA -3′ and 5′- ATAGTTTAGCGGCCGCCTTACACCATTGTTATAGC -3′. The underlined sequence corresponds to Sal1 and Not1 restriction sites respectively. The PCR fragments were digested with Not1 (NEB) and Sal1 (NEB) and ligated with pet28a Not1/Sal1 digested vector. The ligation yielded an N-terminal 6x his-tag fusion that was utilized for purification. The pet28a-3h04 plasmid was then transformed into E. coli BL21D cells by electroporation. The resulting strain was grown in LB broth containing 25 µg/mL of kanamycin at 37°C. When the optical density at 600 nm reached between.5-.6, protein expression was induced by the addition of IPTG (Sigma) to a final concentration of.1 mM and left shaking at 150 rpm overnight at 25°C. The next day, cells were pelleted by centrifugation and frozen at −80°C until needed for purification.

### Purification

4mL of Bugbuster Mastermix Reagent (Novagen) was mixed vigorously with 1 g of cell paste and 5 µL of protease inhibitor cocktail set VII (Calbiochem), and incubated shaking at 4°C for 1 hour. The mixture was then centrifuged to pellet the insoluble debris and the supernatant was mixed with Ni-nitrilotriacetic acid (NTA) agarose resin (Qiagen) equilibrated in wash buffer (50 mM NaH_2_PO_4_ [pH = 8.0], 2 M NaCl, and 2% glycerol) containing 7.5 mM Imidizole and left shaking at 4°C for 1 hour. The column was then washed with 10 column volumes of wash buffer +25 mM imidizole. The bound 3h04 protein was step eluted from the column with wash buffer containing 40–400 mM Imidizole. 3h04 containing fractions were pooled and dialyzed against 50 mM potassium phosphate buffer (monobasic, pH 7.0) containing 10% glycerol. 3h04 protein solution was then concentrated using a 10 kDa cutoff Amicon Ultra-4 centrifugal unit (Millipore). Protein concentration was estimated using the Micro BCA Protein Assay Kit (Thermo).

### Enzyme Assay

Carboxylesterase specific activity was determined by measuring the amount of 4-nitrophenol produced from the hydrolysis of 4-nitrophenyl acetate. One unit (U) of enzyme was defined as the liberation of 1 µmole of 4-nitrophenol per minute. A 100 mM stock solution of 4-nitrophenyl acetate was made by dissolving the substrate in 100% DMSO. The final reaction mixture contained 50 mM 2-(N-Morpholino)ethanesulfonic acid (MES) pH 6.0, 3% DMSO, 1 mM 4-nitrophenyl acetate and enzyme. After pre-incubation for 10 minutes at 25°C, the reaction was initiated by the addition of the substrate. The reaction was monitored by observing the change in absorbance at 405 nm by UV spectrometry (Amersham Ultrospec 3100pro). The molar extinction coefficient used for 4-nitrophenol at 405 nm for the specified conditions was 8,629 M^−1^ cm^−1^.

## Supporting Information

Table S1 Novel structural genomics predictions. The improvement in our method allowed us to make leads to 257 new high-confidence functional predictions of Structural Genomics proteins.
(0.19 MB DOC)

Figure S1 3 EC Performance on Structural Genomics test set. S3A. Accuracy/coverage tradeoffs of ETA network diffusion and nearest neighbor are shown in red and blue circles respectively. Coverage increases as confidence decreases, meaning at 10% coverage we show the accuracy of our 10% most confident predictions. Blue triangle shows the performance of ETA voting. S3B. Performance compared to the top match from a BLAST search of Swiss-prot. Diffusion on an ETA network clearly outperforms BLAST (black circles) at most coverages on this dataset. S3C: Accuracies when the z score cutoff is varied. For each z score in the range, we plot the accuracy of all predictions with that score or higher. Accuracy shows a steep decline after z = 0.4. S3D shows a magnified view of the beginning of the steep decline.
(0.29 MB TIF)

Figure S2 3 EC Performance on Structural Genomics test set. Accuracy/coverage tradeoffs of ETA network diffusion, nearest neighbor, and the top match from a BLAST search against Swiss-prot are shown in red, blue and black circles respectively. Coverage increases as confidence decreases, meaning at 10% coverage we show the accuracy of our 10% most confident predictions. Maximum allowed sequence identity is 100% in 3A, 80% in 3B, 60% in 3C, 40% in 3D and 20% in 3E. Accuracies decline with each removal, but ETA network diffusion maintains higher accuracy at high confidences/low coverage.
(0.44 MB TIF)

Figure S3 Sequence Identity Between Testset Proteins and Their Top BLAST Match. In order to further explore the relationship between sequence identity and prediction accuracy, we have performed a BLAST search against the Swiss-Prot database and show a histogram of the sequence identity between the query protein and its BLAST match with the smallest e-value. The distribution is not normal: most proteins either have a close homolog, or do not display sequence homology with any proteins in the database.
(0.19 MB TIF)

Figure S4 Additional sources of information that lead to correct predictions. In order to better understand the accuracy gains observed with ETA network diffusion, we have performed several comparisons. A & B: We perform network diffusion with (red) and without (purple) negative labels (labels that denote that a protein does not carry a particular function). Including negative labels increases accuracy by 16% and 10.7% for 3 (A) and 4 (B) digit EC predictions respectively, at 50% coverage, suggesting that negative labels are very important for prediction accuracy. All tests were performed on the structural Genomics testset and the 2008 PDB 90 dataset. C: Distance from nodes with correct 3 EC predictions to nodes with and without the same function. For every protein in the testset for which we make a correct prediction, we show the length of the shortest path to nodes with the same (blue) and different (orange) functions, separated by confidence z-score. All infinite distances are ignored. Highly confident predictions tend to be disconnected from the network. Predictions with lower confidence have fewer close connections with the same function and presumably must rely on information from more distant nodes.
(0.34 MB TIF)

## References

[1] Friedberg I (2006). Automated protein function prediction--the genomic challenge.. Brief Bioinform.

[2] Watson JD, Sanderson S, Ezersky A, Savchenko A, Edwards A (2007). Towards Fully Automated Structure-based Function Prediction in Structural Genomics: A Case Study.. Journal of Molecular Biology.

[3] Chandonia J, Brenner SE (2006). The Impact of Structural Genomics: Expectations and Outcomes.. Science.

[4] Hsiao T, Revelles O, Chen L, Sauer U, Vitkup D (2010). Automatic policing of biochemical annotations using genomic correlations.. Nat Chem Biol.

[5] Brenner SE (1999). Errors in genome annotation.. Trends in Genetics.

[6] Schnoes AM, Brown SD, Dodevski I, Babbitt PC (2009). Annotation error in public databases: misannotation of molecular function in enzyme superfamilies. PLoS Comput.. Biol.

[7] Furnham N, Garavelli JS, Apweiler R, Thornton JM (2009). Missing in action: enzyme functional annotations in biological databases.. Nat Chem Biol.

[8] Hennig S, Groth D, Lehrach H (2003). Automated Gene Ontology annotation for anonymous sequence data.. Nucleic Acids Res.

[9] Altschul SF, Gish W, Miller W, Myers EW, Lipman DJ (1990). Basic local alignment search tool.. Journal of Molecular Biology.

[10] Altschul S, Madden T, Schaffer A, Zhang J, Zhang Z (1997). Gapped BLAST and PSI-BLAST: a new generation of protein database search programs.. Nucl Acids Res.

[11] Engelhardt BE, Jordan MI, Muratore KE, Brenner SE (2005). Protein molecular function prediction by Bayesian phylogenomics.. PLoS Comput Biol.

[12] Glaser F, Pupko T, Paz I, Bell RE, Bechor-Shental D (2003). ConSurf: Identification of Functional Regions in Proteins by Surface-Mapping of Phylogenetic Information.. Bioinformatics.

[13] Chiang RA, Sali A, Babbitt PC (2008). Evolutionarily Conserved Substrate Substructures for Automated Annotation of Enzyme Superfamilies.. PLoS Comput Biol.

[14] van Noort V, Snel B, Huynen MA (2003). Predicting gene function by conserved co-expression.. Trends in Genetics.

[15] Nariai N, Kolaczyk ED, Kasif S Probabilistic Protein Function Prediction from Heterogeneous Genome-Wide Data.. PLoS ONE.

[16] Vazquez A, Flammini A, Maritan A, Vespignani A (2003). Global protein function prediction from protein-protein interaction networks.. Nat Biotech.

[17] Chua HN, Sung W, Wong L (2006). Exploiting indirect neighbours and topological weight to predict protein function from protein-protein interactions.. Bioinformatics.

[18] Collins SR, Miller KM, Maas NL, Roguev A, Fillingham J (2007). Functional dissection of protein complexes involved in yeast chromosome biology using a genetic interaction map.. Nature.

[19] Warde-Farley D, Donaldson SL, Comes O, Zuberi K, Badrawi R (2010). The GeneMANIA prediction server: biological network integration for gene prioritization and predicting gene function.. Nucleic Acids Research.

[20] Arakaki A, Huang Y, Skolnick J (2009). EFICAz2: enzyme function inference by a combined approach enhanced by machine learning.. BMC Bioinformatics.

[21] Jaroszewski L, Rychlewski L, Li Z, Li W, Godzik A (2005). FFAS03: a server for profile-profile sequence alignments.. Nucl Acids Res.

[22] Tseng YY, Dundas J, Liang J (2009). Predicting Protein Function and Binding Profile via Matching of Local Evolutionary and Geometric Surface Patterns.. Journal of Molecular Biology.

[23] Redfern OC, Dessailly BH, Dallman TJ, Sillitoe I, Orengo CA (2009). FLORA: A Novel Method to Predict Protein Function from Structure in Diverse Superfamilies.. PLoS Comput Biol.

[24] Pazos F, Sternberg MJE (2004). Automated prediction of protein function and detection of functional sites from structure.. Proceedings of the National Academy of Sciences of the United States of America.

[25] Ferre F, Ausiello G, Zanzoni A, Helmer-Citterich M (2005). Functional annotation by identification of local surface similarities: a novel tool for structural genomics.. BMC Bioinformatics.

[26] Gold ND, Jackson RM (2006). Fold Independent Structural Comparisons of Protein-Ligand Binding Sites for Exploring Functional Relationships.. Journal of Molecular Biology.

[27] Wang K, Samudrala R (2005). FSSA: a novel method for identifying functional signatures from structural alignments.. Bioinformatics.

[28] Sadowski MI, Jones DT (2009). The sequence-structure relationship and protein function prediction.. Curr Opin Struct Biol.

[29] Alexander PA, He Y, Chen Y, Orban J, Bryan PN (2007). The design and characterization of two proteins with 88% sequence identity but different structure and function.. Proceedings of the National Academy of Sciences.

[30] Porter CT, Bartlett GJ, Thornton JM (2004). The Catalytic Site Atlas: a resource of catalytic sites and residues identified in enzymes using structural data.. Nucl Acids Res.

[31] Kristensen DM, Chen BY, Fofanov VY, Ward RM, Lisewski AM (2006). Recurrent use of evolutionary importance for functional annotation of proteins based on local structural similarity.. Protein Sci.

[32] Gherardini PF, Helmer-Citterich M (2008). Structure-based function prediction: approaches and applications.. Briefings in Functional Genomics and Proteomics.

[33] Laskowski RA, Watson JD, Thornton JM (2005). ProFunc: a server for predicting protein function from 3D structure.. Nucl Acids Res.

[34] Laskowski RA, Watson JD, Thornton JM (2005). Protein function prediction using local 3D templates.. J Mol Biol.

[35] Lichtarge O, Bourne HR, Cohen FE (1996). An evolutionary trace method defines binding surfaces common to protein families.. J Mol Biol.

[36] Lichtarge O, Wilkins A (2010). Evolution: a guide to perturb protein function and networks.. Current Opinion in Structural Biology.

[37] Madabushi S, Yao H, Marsh M, Kristensen DM, Philippi A (2002). Structural clusters of evolutionary trace residues are statistically significant and common in proteins.. J Mol Biol.

[38] Mihalek I, Res I, Lichtarge O (2006). Evolutionary and structural feedback on selection of sequences for comparative analysis of proteins.. Proteins.

[39] Wilkins AD, Lua R, Erdin S, Ward RM, Lichtarge O (2010). Sequence and structure continuity of evolutionary importance improves protein functional site discovery and annotation.. Protein Science.

[40] Lichtarge O, Bourne HR, Cohen FE (1996). Evolutionarily conserved Galphabetagamma binding surfaces support a model of the G protein-receptor complex.. Proc Natl Acad Sci U S A.

[41] Yao H, Kristensen DM, Mihalek I, Sowa ME, Shaw C (2003). An accurate, sensitive, and scalable method to identify functional sites in protein structures.. J Mol Biol.

[42] Sowa ME, He W, Wensel TG, Lichtarge O (2000). A regulator of G protein signaling interaction surface linked to effector specificity.. Proc Natl Acad Sci U S A.

[43] Madabushi S, Gross AK, Philippi A, Meng EC, Wensel TG (2004). Evolutionary trace of G protein-coupled receptors reveals clusters of residues that determine global and class-specific functions.. J Biol Chem.

[44] Baameur F, Morgan DH, Yao H, Tran TM, Hammitt RA (2010). Role for the regulator of G-protein signaling homology domain of G protein-coupled receptor kinases 5 and 6 in beta 2-adrenergic receptor and rhodopsin phosphorylation.. Mol Pharmacol.

[45] Bonde MM, Yao R, Ma J, Madabushi S, Haunsø S (2010). An angiotensin II type 1 receptor activation switch patch revealed through evolutionary trace analysis.. Biochem Pharmacol.

[46] Kobayashi H, Ogawa K, Yao R, Lichtarge O, Bouvier M (2009). Functional rescue of beta-adrenoceptor dimerization and trafficking by pharmacological chaperones.. Traffic.

[47] Ribes-Zamora A, Mihalek I, Lichtarge O, Bertuch AA (2007). Distinct faces of the Ku heterodimer mediate DNA repair and telomeric functions.. Nat Struct Mol Biol.

[48] Rajagopalan L, Patel N, Madabushi S, Goddard JA, Anjan V (2006). Essential helix interactions in the anion transporter domain of prestin revealed by evolutionary trace analysis.. J Neurosci.

[49] Shenoy SK, Drake MT, Nelson CD, Houtz DA, Xiao K (2006). beta-arrestin-dependent, G protein-independent ERK1/2 activation by the beta2 adrenergic receptor.. J Biol Chem.

[50] Gu P, Morgan DH, Sattar M, Xu X, Wagner R (2005). Evolutionary trace-based peptides identify a novel asymmetric interaction that mediates oligomerization in nuclear receptors.. J Biol Chem.

[51] Quan X, Denayer T, Yan J, Jafar-Nejad H, Philippi A (2004). Evolution of neural precursor selection: functional divergence of proneural proteins.. Development.

[52] Sowa ME, He W, Slep KC, Kercher MA, Lichtarge O (2001). Prediction and confirmation of a site critical for effector regulation of RGS domain activity.. Nat Struct Biol.

[53] Onrust R, Herzmark P, Chi P, Garcia PD, Lichtarge O (1997). Receptor and beta gamma Binding Sites in the alpha Subunit of the Retinal G Protein Tr ansducin.. Science.

[54] Rodriguez GJ, Yao R, Lichtarge O, Wensel TG (2010). Evolution-guided discovery and recoding of allosteric pathway specificity determinants in psychoactive bioamine receptors.. Proc Natl Acad Sci U S A.

[55] Mihalek I, Res I, Lichtarge O (2004). A family of evolution-entropy hybrid methods for ranking protein residues by importance.. J Mol Biol.

[56] Ward RM, Erdin S, Tran TA, Kristensen DM, Lisewski AM (2008). De-Orphaning the Structural Proteome through Reciprocal Comparison of Evolutionarily Important Structural Features.. PLoS ONE.

[57] Erdin S, Ward RM, Venner E, Lichtarge O (2010). Evolutionary Trace Annotation of Protein Function in the Structural Proteome.. Journal of Molecular Biology.

[58] Marcotte EM, Pellegrini M, Ng H, Rice DW, Yeates TO (1999). Detecting Protein Function and Protein-Protein Interactions from Genome Sequences.. Science.

[59] Hishigaki H, Nakai K, Ono T, Tanigami A, Takagi T (2001). Assessment of prediction accuracy of protein function from protein-protein interaction data.. Yeast.

[60] Levy E, Ouzounis C, Gilks W, Audit B (2005). Probabilistic annotation of protein sequences based on functional classifications.. BMC Bioinformatics.

[61] Bader G, Hogue C (2003). An automated method for finding molecular complexes in large protein interaction networks.. BMC Bioinformatics.

[62] Newman MEJ (2006). Modularity and Community Structure in Networks.. Proceedings of the National Academy of Sciences of the United States of America.

[63] Adamcsek B, Palla G, Farkas IJ, Derenyi I, Vicsek T (2006). CFinder: locating cliques and overlapping modules in biological networks.. Bioinformatics.

[64] Sharan R, Ulitsky I, Shamir R Network-based prediction of protein function.. Mol Syst Biol.

[65] Song J, Singh M (2009). How and when should interactome-derived clusters be used to predict functional modules and protein function?. Bioinformatics.

[66] Deng M, Zhang K, Mehta S, Chen T, Sun F (2003). Prediction of Protein Function Using Protein-Protein Interaction Data.. J Comput Biol.

[67] Nabieva E, Jim K, Agarwal A, Chazelle B, Singh M (2005). Whole-proteome prediction of protein function via graph-theoretic analysis of interaction maps.. Bioinformatics.

[68] Qi Y, Suhail Y, Lin Y, Boeke JD, Bader JS (2008). Finding friends and enemies in an enemies-only network: A graph diffusion kernel for predicting novel genetic interactions and co-complex membership from yeast genetic interactions.. Genome Research.

[69] Shin H, Lisewski AM, Lichtarge O (2007). Graph sharpening plus graph integration: a synergy that improves protein functional classification.. Bioinformatics.

[70] Hu P, Jiang H, Emili A (2010). Predicting protein functions by relaxation labelling protein interaction network.. BMC Bioinformatics.

[71] Zhou D, Bousquet O, Lal TN, Weston J, Schölkopf B (2004). Learning with local and global consistency.. ADVANCES IN NEURAL INFORMATION PROCESSING SYSTEMS 16.

[72] Tsuda K, Shin H, Schölkopf B (2005). Fast protein classification with multiple networks.. Bioinformatics.

[73] Westbrook J, Feng Z, Chen L, Yang H, Berman HM (2003). The Protein Data Bank and structural genomics.. Nucleic Acids Research.

[74] Brenner SE (2001). A tour of structural genomics.. Nat Rev Genet.

[75] Redfern OC, Harrison A, Dallman T, Pearl FMG, Orengo CA (2007). CATHEDRAL: A Fast and Effective Algorithm to Predict Folds and Domain Boundaries from Multidomain Protein Structures.. PLoS Comput Biol.

[76] Laskowski RA, Watson JD, Thornton JM (2005). Protein Function Prediction Using Local 3D Templates.. Journal of Molecular Biology.

[77] She R, Chu JS, Wang K, Pei J, Chen N (2009). genBlastA: Enabling BLAST to identify homologous gene sequences.. Genome Research.

[78] The UniProt Consortium (2010). The Universal Protein Resource (UniProt) in 2010.. Nucl Acids Res.

[79] Gill SR, Fouts DE, Archer GL, Mongodin EF, DeBoy RT (2005). Insights on Evolution of Virulence and Resistance from the Complete Genome Analysis of an Early Methicillin-Resistant Staphylococcus aureus Strain and a Biofilm-Producing Methicillin-Resistant Staphylococcus epidermidis Strain.. J Bacteriol.

[80] Greene LH, Lewis TE, Addou S, Cuff A, Dallman T (2007). The CATH domain structure database: new protocols and classification levels give a more comprehensive resource for exploring evolution.. Nucleic Acids Res.

[81] Holm L, Kaariainen S, Rosenstrom P, Schenkel A (2008). Searching protein structure databases with DaliLite v.3.. Bioinformatics.

[82] Byun J, Rhee J, Kim N, Yoon J, Kim D (2007). Crystal structure of hyperthermophilic esterase EstE1 and the relationship between its dimerization and thermostability properties.. BMC Structural Biology.

[83] Rhee J, Ahn D, Kim Y, Oh J (2005). New Thermophilic and Thermostable Esterase with Sequence Similarity to the Hormone-Sensitive Lipase Family, Cloned from a Metagenomic Library.. Appl Environ Microbiol.

[84] Krisch K (1966). Reaction of a microsomal esterase from hog-liver with diethyl rho-nitrophenyl phosphate.. Biochim Biophys Acta.

[85] Wu PC, Liu YH, Wang ZY, Zhang XY, Li H (2006). Molecular cloning, purification, and biochemical characterization of a novel pyrethroid-hydrolyzing esterase from Klebsiella sp. strain ZD112.. J Agric Food Chem.

[86] Ross MK, Borazjani A, Edwards CC, Potter PM (2006). Hydrolytic metabolism of pyrethroids by human and other mammalian carboxylesterases. Biochem.. Pharmacol.

[87] Mukherjee JJ, Jay FT, Choy PC (1993). Purification, characterization and modulation of a microsomal carboxylesterase in rat liver for the hydrolysis of acyl-CoA.. Biochem J.

[88] Capra JA, Laskowski RA, Thornton JM, Singh M, Funkhouser TA (2009). Predicting Protein Ligand Binding Sites by Combining Evolutionary Sequence Conservation and 3D Structure.. PLoS Comput Biol.

[89] Lee I, Lehner B, Crombie C, Wong W, Fraser AG (2008). A single gene network accurately predicts phenotypic effects of gene perturbation in Caenorhabditis elegans. Nat.. Genet.

[90] Lee I, Date SV, Adai AT, Marcotte EM (2004). A probabilistic functional network of yeast genes.. Science.

[91] Almonacid DE, Yera ER, Mitchell JBO, Babbitt PC (2010). Quantitative Comparison of Catalytic Mechanisms and Overall Reactions in Convergently Evolved Enzymes: Implications for Classification of Enzyme Function.. PLoS Comput Biol.

[92] Atkinson HJ, Babbitt PC (2009). An Atlas of the Thioredoxin Fold Class Reveals the Complexity of Function-Enabling Adaptations.. PLoS Comput Biol.

[93] Copley SD (2003). Enzymes with extra talents: moonlighting functions and catalytic promiscuity.. Current Opinion in Chemical Biology.

[94] Glazer DS, Radmer RJ, Altman RB (2009). Improving Structure-Based Function Prediction Using Molecular Dynamics.. Structure.

[95] Barrell D, Dimmer E, Huntley RP, Binns D, O'Donovan C (2009). The GOA database in 2009--an integrated Gene Ontology Annotation resource.. Nucleic Acids Res.

[96] Larkin M, Blackshields G, Brown N, Chenna R, McGettigan P (2007). Clustal W and Clustal X version 2.0.. Bioinformatics.

[97] Pandit SB, Brylinski M, Zhou H, Gao M, Arakaki AK (2010). PSiFR: an integrated resource for prediction of protein structure and function.. Bioinformatics.

