# Supplementary material for: Accurate Protein Structure Annotation through Competitive Diffusion of Enzymatic Functions over a Network of Local Evolutionary Similarities
Source: PLoS One. 2010 Dec 13;5(12):e14286. doi: 10.1371/journal.pone.0014286 (PMC3001439; doi:10.1371/journal.pone.0014286)
Supplement: Table S1 — Novel structural genomics predictions. The improvement in our method allowed us to make leads to 257 new high-confidence functional predictions of Structural Genomics proteins. (0.19 MB DOC) [file pone.0014286.s001.doc]

Table S1: Predictions

| 2awpA | 1.15.1.1 | 10.58666 |
| --- | --- | --- |
| 2gw2A | 5.2.1.8 | 9.407802 |
| 3h1sA | 1.15.1.1 | 8.84157 |
| 2fu0A | 5.2.1.8 | 8.723306 |
| 2b71A | 5.2.1.8 | 8.702754 |
| 1z2uA | 6.3.2.19 | 5.907579 |
| 3krsA | 5.3.1.1 | 5.746931 |
| 2yxgA | 4.2.1.52 | 5.19338 |
| 3e95A | 6.3.2.19 | 4.969848 |
| 3gvfA | 3.6.1.1 | 4.604141 |
| 2o25C | 6.3.2.19 | 4.322212 |
| 3ktcA | 5.3.1.5 | 4.187508 |
| 3g2gA | 2.7.1.40 | 4.176847 |
| 3gr4A | 2.7.1.40 | 4.174963 |
| 3h6oA | 2.7.1.40 | 4.172795 |
| 2ef0A | 2.1.3.3 | 4.162439 |
| 2f4zA | 6.3.2.19 | 4.06259 |
| 3gqyA | 2.7.1.40 | 4.017372 |
| 2yr1A | 4.2.1.10 | 3.605515 |
| 3k5kA | 2.1.1.43 | 3.462737 |
| 2pbcA | 5.2.1.8 | 3.333588 |
| 1twlA | 3.6.1.1 | 3.270969 |
| 1npdA | 1.1.1.25 | 3.23709 |
| 3gkbA | 4.2.1.17 | 3.100209 |
| 3dr3A | 1.2.1.38 | 3.096379 |
| 1jn1A | 4.6.1.12 | 3.088211 |
| 3mdyD | 5.2.1.8 | 3.071486 |
| 3mdyB | 5.2.1.8 | 3.070955 |
| 1xq9A | 5.4.2.1 | 2.986382 |
| 3czhA | 1.14.14.1 | 2.946625 |
| 3l12A | 3.1.4.46 | 2.896428 |
| 2a7vA | 2.1.2.1 | 2.868092 |
| 2hteA | 2.5.1.16 | 2.844468 |
| 2o1zA | 1.17.4.1 | 2.843847 |
| 2oczA | 4.2.1.10 | 2.788775 |
| 3dl9A | 1.14.14.1 | 2.763467 |
| 3c6gA | 1.14.14.1 | 2.754617 |
| 2ocdA | 3.5.1.1 | 2.477973 |
| 2yxeA | 2.1.1.77 | 2.426371 |
| 2g17A | 1.2.1.38 | 2.411218 |
| 3ic9A | 1.8.1.4 | 2.407308 |
| 2gwnA | 3.5.2.2 | 2.367561 |
| 2fo3A | 6.3.2.19 | 2.259226 |
| 1p1mA | 3.5.4.3 | 2.252424 |
| 3ijpA | 1.3.1.26 | 2.203085 |
| 3exaA | 2.5.1.8 | 2.166634 |
| 2qgnA | 2.5.1.8 | 2.151311 |
| 2onuA | 6.3.2.19 | 2.149313 |
| 1tqxA | 5.1.3.1 | 2.089528 |
| 2gryA | 3.6.4.4 | 2.078838 |
| 2hehA | 3.6.4.4 | 2.066919 |
| 1uf9A | 2.7.1.24 | 2.054443 |
| 2repA | 3.6.4.4 | 2.05406 |
| 3b6vA | 3.6.4.4 | 2.024553 |
| 3h04A | 3.1.1.1 | 1.925791 |
| 3gbjA | 3.6.4.4 | 1.867902 |
| 1syrA | 1.8.1.9 | 1.819901 |
| 1vl0A | 1.1.1.133 | 1.752772 |
| 1o12A | 3.5.1.25 | 1.74065 |
| 1xi6A | 3.1.3.25 | 1.706668 |
| 2amuA | 1.15.1.2 | 1.705625 |
| 3cbwA | 3.2.1.78 | 1.70265 |
| 2fuvA | 5.4.2.2 | 1.675074 |
| 1zbrA | 3.5.3.12 | 1.664785 |
| 1t9kA | 5.3.1.23 | 1.66244 |
| 3innA | 6.3.2.1 | 1.655932 |
| 2h2yA | 6.3.2.19 | 1.651023 |
| 3groA | 3.1.2.22 | 1.639531 |
| 2ajpA | 2.7.1.35 | 1.638641 |
| 2ah6A | 2.5.1.17 | 1.636873 |
| 1rtyA | 2.5.1.17 | 1.620346 |
| 2ar0A | 2.1.1.72 | 1.613998 |
| 2bddA | 2.7.8.7 | 1.601906 |
| 2f6rA | 2.7.1.24 | 1.598422 |
| 1nr9A | 5.3.3.10 | 1.597408 |
| 1y89A | 3.1.1.31 | 1.574193 |
| 2a6pA | 5.4.2.1 | 1.572665 |
| 3devA | 3.6.1.1 | 1.569174 |
| 1whcA | 3.1.2.15 | 1.522696 |
| 2ig7A | 2.7.1.32 | 1.513589 |
| 1ou0A | 5.4.1.2 | 1.479642 |
| 1vekA | 3.1.2.15 | 1.468502 |
| 2a0mA | 3.5.3.11 | 1.459861 |
| 3b6uA | 3.6.4.4 | 1.456912 |
| 1ylkA | 4.2.1.1 | 1.455793 |
| 3ks6A | 3.1.4.46 | 1.426547 |
| 1yf9A | 6.3.2.19 | 1.42636 |
| 2nztA | 2.7.1.1 | 1.423593 |
| 3ewbX | 2.3.3.13 | 1.412106 |
| 1npyA | 1.1.1.25 | 1.388838 |
| 3h0kA | 2.7.4.3 | 1.378969 |
| 2fg5A | 3.6.5.2 | 1.336395 |
| 1nogA | 2.5.1.17 | 1.328049 |
| 3db2A | 1.1.1.18 | 1.292476 |
| 3ec7A | 1.1.1.18 | 1.283077 |
| 3ecsA | 5.3.1.23 | 1.274469 |
| 2q3rA | 1.3.1.42 | 1.192445 |
| 1xccA | 1.11.1.15 | 1.129786 |
| 1ilvA | 3.1.3.5 | 1.073779 |
| 1rxdA | 3.1.3.48 | 1.02692 |
| 1xv2A | 4.1.1.5 | 1.026721 |
| 1xi3A | 2.5.1.3 | 1.023457 |
| 2i5tA | 2.3.2.4 | 1.015678 |
| 2q53A | 2.3.2.4 | 1.014853 |
| 2hi1A | 1.1.1.262 | 1.000427 |
| 1xriA | 3.1.3.48 | 0.998999 |
| 2o1qA | 1.13.11.50 | 0.998148 |
| 2pq8A | 2.3.1.48 | 0.98941 |
| 1xhoA | 5.4.99.5 | 0.988295 |
| 3cbqA | 3.6.5.2 | 0.987982 |
| 2q3uA | 3.5.3.12 | 0.986574 |
| 2amxA | 3.5.4.4 | 0.98447 |
| 1jovA | 5.1.3.15 | 0.982119 |
| 3gftA | 3.6.5.2 | 0.977877 |
| 3k67A | 4.2.1.17 | 0.965702 |
| 1x87A | 4.2.1.49 | 0.96565 |
| 3daxA | 5.3.99.4 | 0.963101 |
| 3feuA | 1.8.4.2 | 0.961923 |
| 2a9fA | 1.1.1.38 | 0.96186 |
| 3l60A | 2.3.1.12 | 0.961482 |
| 2px4A | 2.7.7.48 | 0.960251 |
| 2ffiA | 3.1.1.57 | 0.958139 |
| 1wkcA | 6.3.3.2 | 0.955499 |
| 1v5tA | 3.1.2.15 | 0.95243 |
| 2ekzA | 5.4.2.1 | 0.946048 |
| 1vraB | 2.3.1.35 | 0.939703 |
| 2r8bA | 3.1.1.1 | 0.939232 |
| 3do8A | 2.7.7.3 | 0.934608 |
| 1x5xA | 3.1.3.48 | 0.930808 |
| 1zghA | 2.1.2.9 | 0.93068 |
| 2be3A | 2.7.6.5 | 0.927037 |
| 1yreA | 2.3.1.128 | 0.924511 |
| 3fj1A | 2.6.1.16 | 0.924311 |
| 3jygA | 4.6.1.10 | 0.92129 |
| 2gtaA | 3.6.1.19 | 0.920859 |
| 3cswA | 2.6.1.42 | 0.920304 |
| 3ffjA | 3.2.1.20 | 0.919852 |
| 3dcdA | 5.1.3.15 | 0.91973 |
| 1x77A | 1.7.1.6 | 0.917113 |
| 2qiwA | 4.1.3.30 | 0.916855 |
| 3gv1A | 5.3.4.1 | 0.916848 |
| 3ci6A | 2.7.3.9 | 0.915736 |
| 1xviA | 3.1.3.70 | 0.912748 |
| 2e5fA | 3.5.99.6 | 0.911398 |
| 2hiaA | 5.4.2.1 | 0.911018 |
| 2cqzA | 3.1.3.5 | 0.909761 |
| 1sqeA | 1.14.99.3 | 0.909208 |
| 2hrzA | 1.1.1.103 | 0.907571 |
| 2nrhA | 2.7.1.33 | 0.901162 |
| 3gmsA | 1.3.1.38 | 0.900215 |
| 2hjsA | 1.2.1.11 | 0.899677 |
| 1ydfA | 3.1.3.74 | 0.899525 |
| 2gl6A | 2.7.3.2 | 0.898714 |
| 3g17A | 1.1.1.169 | 0.898658 |
| 3hdjA | 1.4.1.1 | 0.898371 |
| 3hgbA | 1.4.4.2 | 0.896883 |
| 2folA | 3.6.5.2 | 0.893012 |
| 2pktA | 3.1.3.48 | 0.890191 |
| 3b8bA | 3.1.3.25 | 0.889128 |
| 1yemA | 4.6.1.1 | 0.888842 |
| 1rttA | 1.7.1.6 | 0.886517 |
| 1sc0A | 3.1.2.23 | 0.885993 |
| 3ebvA | 3.2.1.14 | 0.885124 |
| 3fvwA | 1.7.1.6 | 0.881551 |
| 2rbcA | 2.7.1.15 | 0.876288 |
| 3fk2A | 3.6.5.2 | 0.874076 |
| 1wviA | 3.1.3.74 | 0.871305 |
| 2dc4A | 4.6.1.1 | 0.870238 |
| 1uemA | 3.1.3.48 | 0.865976 |
| 2b6eA | 3.1.2.23 | 0.864251 |
| 2yzjA | 3.5.4.13 | 0.863709 |
| 1ydmA | 6.3.3.2 | 0.862522 |
| 3dl2A | 1.1.1.27 | 0.861856 |
| 3fbgA | 1.6.5.5 | 0.861494 |
| 2o1oA | 2.5.1.10 | 0.859594 |
| 1xr4A | 4.1.3.6 | 0.858336 |
| 3cj8A | 2.3.1.117 | 0.855886 |
| 2acaA | 4.6.1.1 | 0.851663 |
| 2qmlA | 2.3.1.82 | 0.850056 |
| 1xeaA | 1.1.1.18 | 0.849885 |
| 1yv9A | 3.1.3.74 | 0.848696 |
| 2zdcA | 3.5.4.13 | 0.848 |
| 2gpyA | 2.1.1.6 | 0.847206 |
| 2kkqA | 2.7.11.18 | 0.84542 |
| 3kd3A | 3.1.3.3 | 0.843447 |
| 3k25A | 5.1.3.15 | 0.842302 |
| 2dr3A | 2.7.1.37 | 0.837047 |
| 3e18A | 1.1.99.28 | 0.834645 |
| 1rtwA | 2.5.1.2 | 0.826861 |
| 1wf7A | 3.1.3.48 | 0.822126 |
| 1ys9A | 3.1.3.74 | 0.820455 |
| 3labA | 4.1.2.14 | 0.81713 |
| 3n2cA | 3.5.4.2 | 0.814573 |
| 1vdwA | 3.1.3.25 | 0.813539 |
| 3be7A | 3.5.4.2 | 0.813159 |
| 2qltA | 3.1.3.18 | 0.806894 |
| 2ka7A | 1.4.4.2 | 0.804993 |
| 3eo4A | 2.3.1.1 | 0.802435 |
| 3h1nA | 5.3.99.2 | 0.79708 |
| 1tt7A | 1.6.5.5 | 0.793676 |
| 2qgyA | 4.2.1.90 | 0.791429 |
| 2rexD | 3.6.5.2 | 0.789064 |
| 3c5eA | 6.2.1.2 | 0.788296 |
| 1vp2A | 3.6.1.19 | 0.783438 |
| 2ftzA | 2.5.1.10 | 0.781763 |
| 1kq3A | 1.1.1.6 | 0.781566 |
| 1y9eA | 1.6.5.5 | 0.77862 |
| 2idaA | 3.1.2.15 | 0.776762 |
| 1z90A | 2.7.7.9 | 0.77246 |
| 2egxA | 2.7.2.8 | 0.771502 |
| 1o5zA | 6.3.2.17 | 0.768714 |
| 2g6bA | 3.6.5.2 | 0.766331 |
| 2df8A | 3.5.99.6 | 0.762366 |
| 2decA | 3.5.99.6 | 0.758834 |
| 3e5zA | 3.1.1.17 | 0.75838 |
| 2cq9A | 1.20.4.1 | 0.755907 |
| 2pq0A | 3.1.3.70 | 0.745314 |
| 2qyhA | 3.1.3.70 | 0.743603 |
| 3flvA | 5.3.3.8 | 0.741828 |
| 2hxvA | 3.5.4.26 | 0.722152 |
| 2q47A | 3.1.3.48 | 0.714484 |
| 2a35A | 1.1.1.2 | 0.704232 |
| 3dljA | 3.4.13.18 | 0.702443 |
| 1x4xA | 2.7.1.112 | 0.700264 |
| 2il1A | 3.6.5.2 | 0.69531 |
| 2oqtA | 2.7.1.69 | 0.693425 |
| 2gf9A | 3.6.5.2 | 0.693377 |
| 1spkA | 2.7.10.2 | 0.684868 |
| 1x5aA | 3.1.3.48 | 0.682902 |
| 1xa0A | 1.6.5.5 | 0.679989 |
| 2eg0A | 4.2.1.1 | 0.679907 |
| 3bfnA | 3.6.4.4 | 0.669176 |
| 1pw5A | 3.1.3.74 | 0.666921 |
| 1xhdA | 4.2.1.1 | 0.656168 |
| 2gqfA | 1.18.6.1 | 0.653816 |
| 2daiA | 3.1.2.15 | 0.646377 |
| 3d6kA | 4.4.1.14 | 0.643615 |
| 3mdyC | 2.7.1.37 | 0.642903 |
| 2npnA | 2.1.1.130 | 0.642373 |
| 2gswA | 1.7.1.6 | 0.628648 |
| 2creA | 2.7.1.112 | 0.628525 |
| 3mdyA | 2.7.1.37 | 0.627606 |
| 1ujdA | 3.1.3.48 | 0.625083 |
| 1z7aA | 3.5.2.5 | 0.62122 |
| 2b3mA | 4.2.1.17 | 0.620278 |
| 3kl2A | 3.5.1.59 | 0.6104 |
| 1xi9A | 2.6.1.1 | 0.603794 |
| 3ez1A | 4.4.1.13 | 0.59503 |
| 3eo3A | 5.1.3.14 | 0.592597 |
| 1t5jA | 3.2.1.143 | 0.577701 |
| 1vh5A | 3.1.2.23 | 0.551432 |
| 1vi8A | 3.1.2.23 | 0.54771 |
| 1zkpA | 3.1.26.11 | 0.538502 |
| 1kq4A | 2.1.1.148 | 0.533091 |
| 1vb7A | 3.1.3.48 | 0.529281 |
| 3ixcA | 4.2.1.1 | 0.522297 |
| 2fe7A | 2.3.1.57 | 0.502228 |
