# Supplementary figures and images for: Accurate Protein Structure Annotation through Competitive Diffusion of Enzymatic Functions over a Network of Local Evolutionary Similarities
Source: PLoS One. 2010 Dec 13;5(12):e14286. doi: 10.1371/journal.pone.0014286 (PMC3001439; doi:10.1371/journal.pone.0014286)

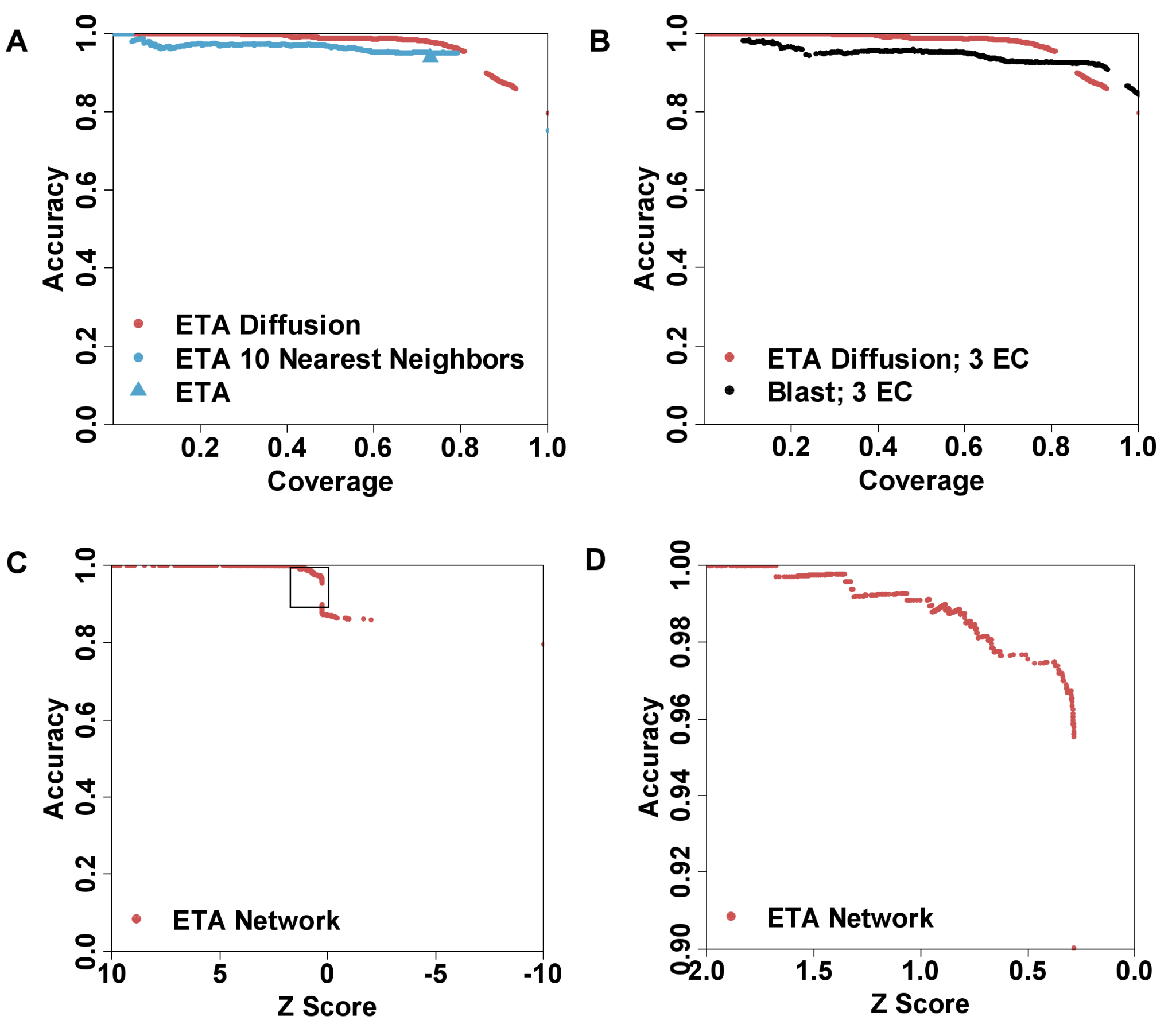

Supplement: Figure S1 — 3 EC Performance on Structural Genomics test set. S3A. Accuracy/coverage tradeoffs of ETA network diffusion and nearest neighbor are shown in red and blue circles respectively. Coverage increases as confidence decreases, meaning at 10% coverage we show the accuracy of our 10% most confident predictions. Blue triangle shows the performance of ETA voting. S3B. Performance compared to the top match from a BLAST search of Swiss-prot. Diffusion on an ETA network clearly outperforms BLAST (black circles) at most coverages on this dataset. S3C: Accuracies when the z score cutoff is varied. For each z score in the range, we plot the accuracy of all predictions with that score or higher. Accuracy shows a steep decline after z = 0.4. S3D shows a magnified view of the beginning of the steep decline. (0.29 MB TIF) [file pone.0014286.s002.tif]

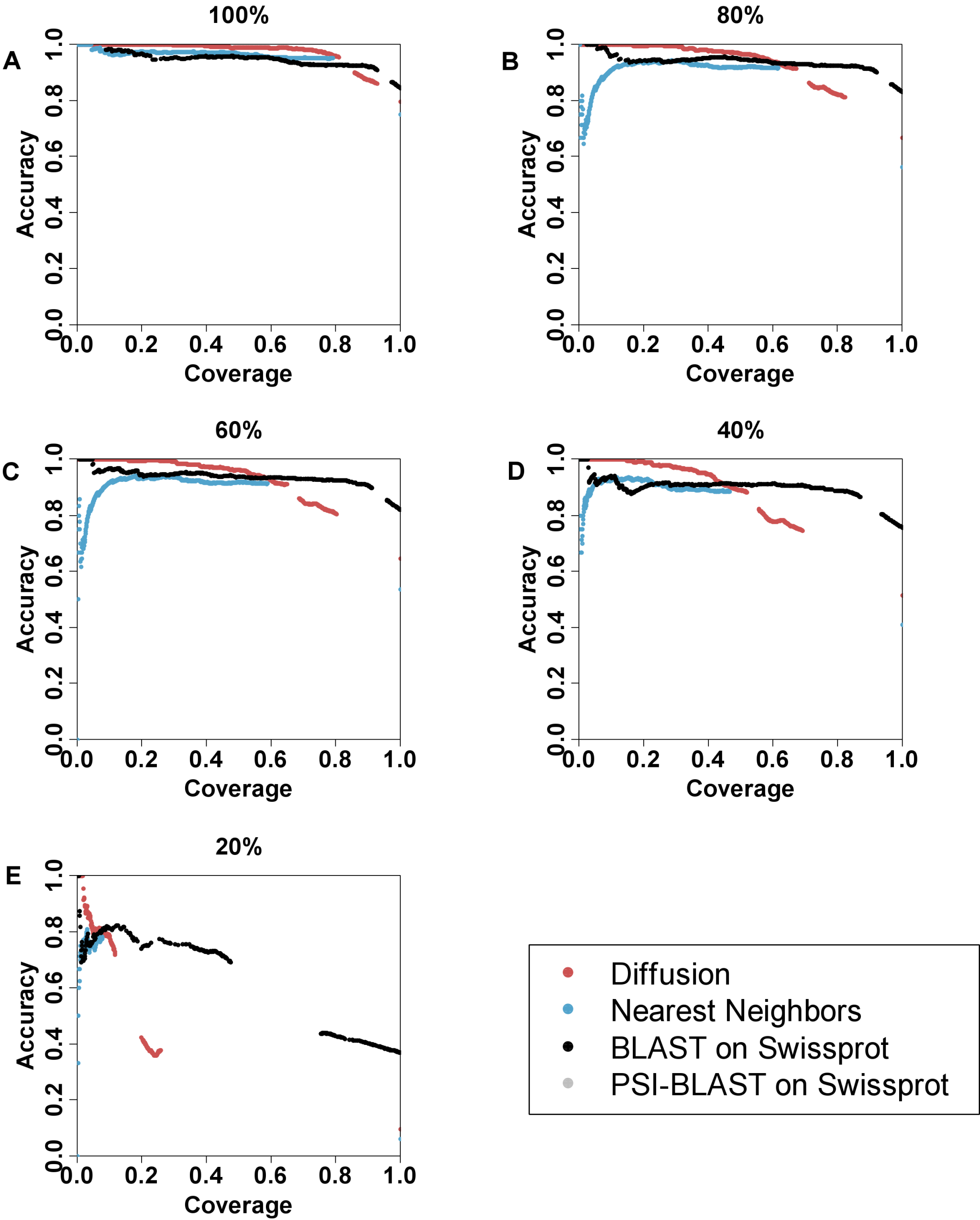

Supplement: Figure S2 — 3 EC Performance on Structural Genomics test set. Accuracy/coverage tradeoffs of ETA network diffusion, nearest neighbor, and the top match from a BLAST search against Swiss-prot are shown in red, blue and black circles respectively. Coverage increases as confidence decreases, meaning at 10% coverage we show the accuracy of our 10% most confident predictions. Maximum allowed sequence identity is 100% in 3A, 80% in 3B, 60% in 3C, 40% in 3D and 20% in 3E. Accuracies decline with each removal, but ETA network diffusion maintains higher accuracy at high confidences/low coverage. (0.44 MB TIF) [file pone.0014286.s003.tif]

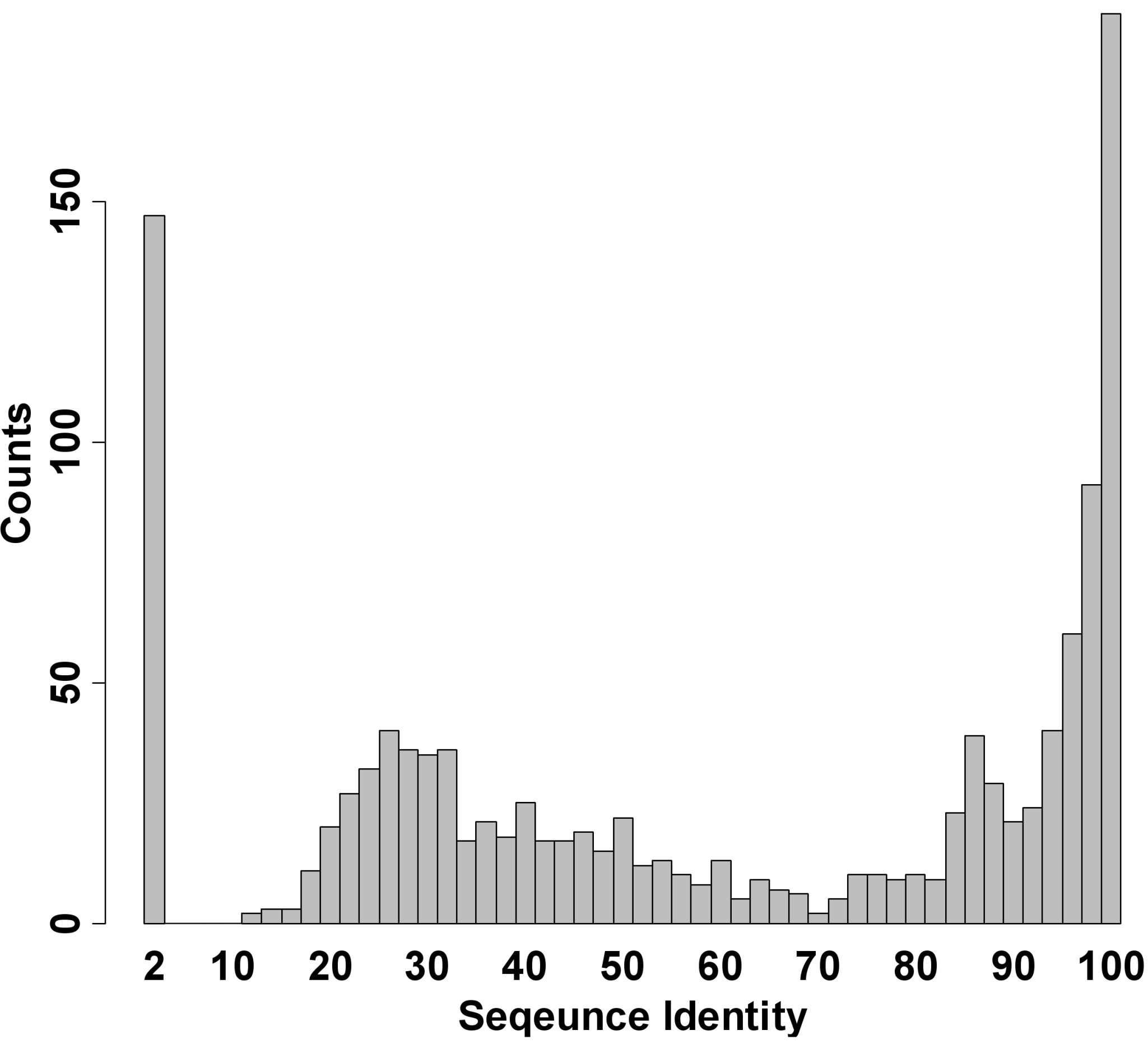

Supplement: Figure S3 — Sequence Identity Between Testset Proteins and Their Top BLAST Match. In order to further explore the relationship between sequence identity and prediction accuracy, we have performed a BLAST search against the Swiss-Prot database and show a histogram of the sequence identity between the query protein and its BLAST match with the smallest e-value. The distribution is not normal: most proteins either have a close homolog, or do not display sequence homology with any proteins in the database. (0.19 MB TIF) [file pone.0014286.s004.tif]

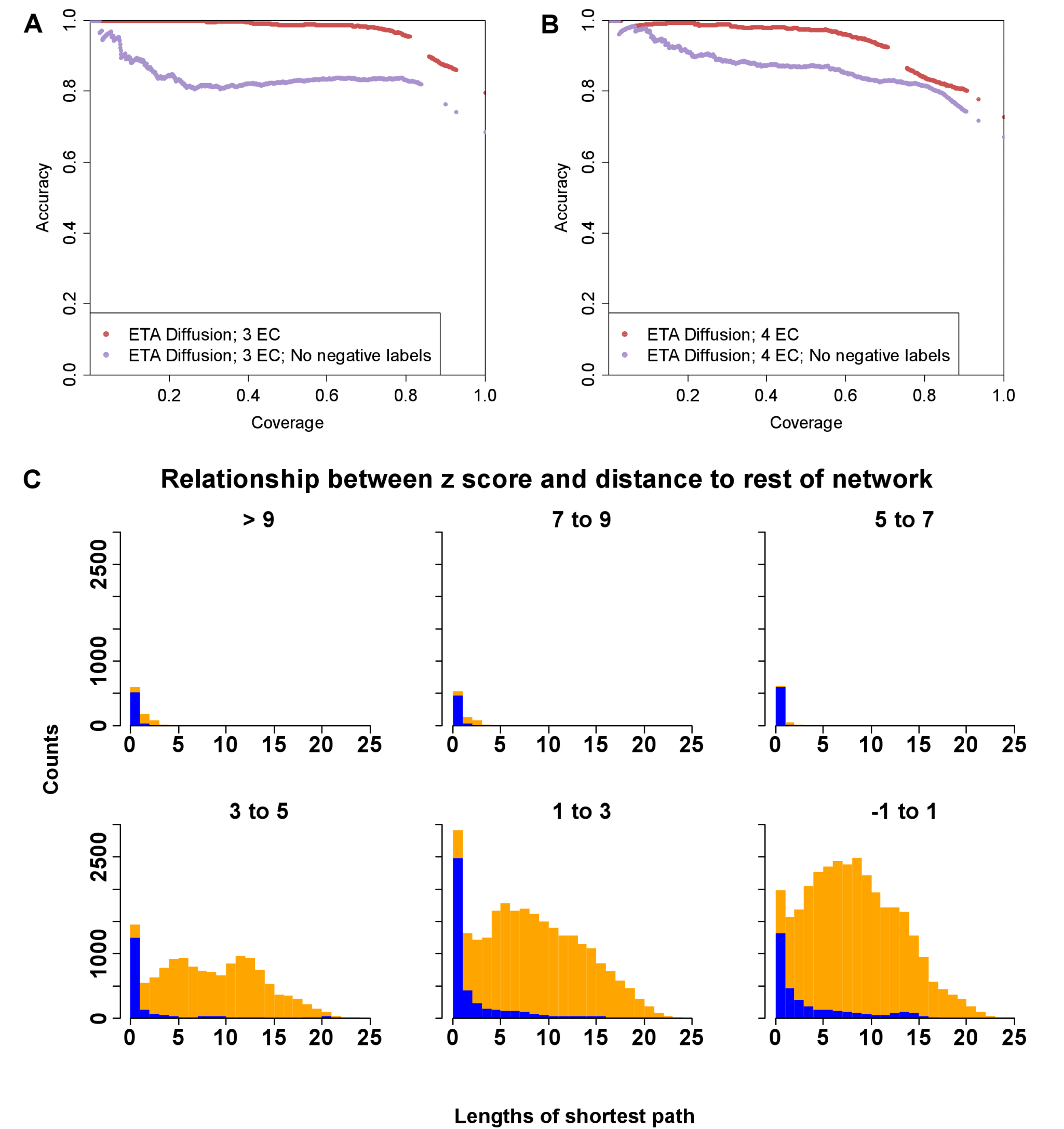

Supplement: Figure S4 — Additional sources of information that lead to correct predictions. In order to better understand the accuracy gains observed with ETA network diffusion, we have performed several comparisons. A & B: We perform network diffusion with (red) and without (purple) negative labels (labels that denote that a protein does not carry a particular function). Including negative labels increases accuracy by 16% and 10.7% for 3 (A) and 4 (B) digit EC predictions respectively, at 50% coverage, suggesting that negative labels are very important for prediction accuracy. All tests were performed on the structural Genomics testset and the 2008 PDB 90 dataset. C: Distance from nodes with correct 3 EC predictions to nodes with and without the same function. For every protein in the testset for which we make a correct prediction, we show the length of the shortest path to nodes with the same (blue) and different (orange) functions, separated by confidence z-score. All infinite distances are ignored. Highly confident predictions tend to be disconnected from the network. Predictions with lower confidence have fewer close connections with the same function and presumably must rely on information from more distant nodes. (0.34 MB TIF) [file pone.0014286.s005.tif]
